# Classification of Compressed Remote Sensing Multispectral Images via Convolutional Neural Networks

**DOI:** 10.3390/jimaging6040024

**Published:** 2020-04-18

**Authors:** Michalis Giannopoulos, Anastasia Aidini, Anastasia Pentari, Konstantina Fotiadou, Panagiotis Tsakalides

**Affiliations:** 1 Signal Processing Lab (SPL), Institute of Computer Science, Foundation for Research and Technology-Hellas (FORTH), 70013 Crete, Greece; aidini@csd.uoc.gr (A.A.); anpentari@gmail.com (A.P.); kfot@ics.forth.gr (K.F.); tsakalid@ics.forth.gr (P.T.); 2 Computer Science Department, University of Crete, 70013 Crete, Greece

**Keywords:** multispectral image classification, deep learning, convolutional neural networks, residual learning, compression, quantization, tensor unfoldings, nuclear norm

## Abstract

Multispectral sensors constitute a core Earth observation image technology generating massive high-dimensional observations. To address the communication and storage constraints of remote sensing platforms, lossy data compression becomes necessary, but it unavoidably introduces unwanted artifacts. In this work, we consider the encoding of multispectral observations into high-order tensor structures which can naturally capture multi-dimensional dependencies and correlations, and we propose a resource-efficient compression scheme based on quantized low-rank tensor completion. The proposed method is also applicable to the case of missing observations due to environmental conditions, such as cloud cover. To quantify the performance of compression, we consider both typical image quality metrics as well as the impact on state-of-the-art deep learning-based land-cover classification schemes. Experimental analysis on observations from the ESA Sentinel-2 satellite reveals that even minimal compression can have negative effects on classification performance which can be efficiently addressed by our proposed recovery scheme.

## 1. Introduction

The domain of *Remote Sensing* (RS) focuses on monitoring the physical characteristics of a target area by measuring the reflected and emitted radiation from a distance, in contrast to in situ observations which require on-site measuring devices. To achieve this objective, airborne and spaceborne *Multispectral* (MS) and *Hyperspectral* (HS) sensors, capable of observing an extended region of the electromagnetic spectrum, are used in order to detect and classify objects on Earth, exploiting the unique spectral profiles of different materials [1]. While the acquisition of a large number of observations is critical for Earth observation, as it provides valuable spatial and spectral information, the copious amounts of data collected from high-resolution sensors over extended time periods that can be formulated by means of high-dimensional data structures, known as tensors, introduce considerable challenges in terms of data storage and data transfer [2]. Especially in RS cases, MS images that are modeled as a third-order tensor defined by two spatial and one spectral variable, are collected on satellites or unmanned aerial vehicles and need to be transferred to the ground-based stations. For that purpose, compression of the acquired MS images is mandatory, in order to reduce bandwidth and increase the system lifetime [3].

To that end, a number of lossless and lossy compression approaches have been proposed, including transform coding methods, such as the *Discrete Cosine Transform* and the *Discrete Wavelet Transform* ( DWT) [4]. While lossless compression schemes are designed so that no information is lost during compression, they typically offer moderate compression rates, in the range of 1.5:1 to 3:1. On the other hand, lossy compression techniques can achieve much higher compression rates, in excess of 20:1, primarily by mapping a range of values to values from discrete sets-a process known as quantization-which can be applied to the original pixels or their representation in some transform domain.

Once the massive amounts of observations acquired by modern instruments, including satellites such as ESA’s Sentinel 2 and NASA’s Landsat, are transferred to ground-station, methodologies that will allow the automation of classification tasks are necessary. To that end, several *Machine Learning* (ML) algorithms have been employed by the RS community, including *Support Vector Machines* (SVMs) [5] and *Random Forests* (RFs) [6]. In the past five years, *Deep Learning* (DL) and in particular architectures such as *Convolutional Neural Networks* (CNN) have attracted considerable attention due to their significant success in various RS analysis tasks, including multi-label classification [7], land cover classification [8], object detection [9] and image retrieval [10], among others. Specifically, CNNs pass filters, widely known as kernels, over the raw image data aiming to exploit the underlying correlations among existing pixels to design as representative and compact features as possible. Most interestingly, CNNs are not fixed but instead learned through the training process of the network [11].

Since satellite imagery data depend directly on spacecraft operations, the *Launch and Early Orbit Phase* (LEOP) of space missions is always treated with great concern and caution. During LEOP, operations staff have to activate, monitor and control the various subsystems of the satellite, including the deployment of any satellite appendages (e.g., antennas, solar array, reflector, etc.), and undertake critical orbit and attitude control maneuvers. Engineering calibration (e.g., platform, instrument, and processor calibration) and geophysical validation (e.g., collection, quality control and archiving of correlative data) are crucial activities associated with this stage. Unfortunately, even though the launch phase is designed to provide high-quality reference data for the training of the ML algorithms, during test phase the online monitoring data are in most cases noise-infected (e.g., compressed) versions of the reference ones. This has an adverse effect on the ML task as the models are tested with data with significantly different statistical distributions than those during training. Motivated by this real-world problem, we conducted the present study with the intention of designing and providing a system capable of mitigating the effects of the quantization and subsampling operations.

In this paper, the effect of compression on the classification task of MS images is tackled via the notion of CNNs using a tensor recovery algorithm. More specifically, a recently released MS dataset is used to train and evaluate the performance of the employed CNN with respect to the crucial parameter of the samples it was trained with. Furthermore, a recovery algorithm of the real-valued measurements on high-dimensional data from its quantized and possibly subsampled values-due to noise or communication failures-is proposed in order to further process more efficiently the observed measurements. In particular, we consider a constrained maximum likelihood estimation of a low-rank tensor, in combination with a quantization and statistical measurement model, taking into account the quantization bin boundaries and the existence of possible noise. Subsequently, we quantify the effects of the aforementioned quantization, subsampling, and recovery processes, solely and in conjunction, in the evaluation process of the network in order to investigate its response under hostile real-world regimes. Experimental results on real satellite MS images demonstrate that when trained with a decent number of samples, CNNs can perform quite well in the MS image classification task. Moreover, direct processing with the quantized or subsampled measurements, rather than recovering their real values, ends up with a significantly higher error, indicating the efficacy of the proposed method.

All in all, the key contributions of this paper can be summarized as follows:

- Usage of CNNs (pre-trained and from-scratch models) to tackle the (quantized vs. original) MS image classification task of a recently released dataset.
- Investigation of the dataset size with which a CNN should be trained to efficiently classify real satellite MS images.
- Exploration of the effect of the quantization and subsampling processes on the MS image classification task.
- Provision of a recovery algorithm of the real-valued measurements on high-dimensional data from their quantized and possibly corrupted observations.
- Quantification of the classification scheme’s performance on real quantized & subsampled recovered satellite MS images, highlighting the clear merits when it operates on the recovered images vis-à-vis their quantized counterparts.

## 2. Related Work

In this section, we review the state-of-the-art in terms of quantifying and optimizing the impact of MS/HS compression as well as sampling on subsequent analysis tasks such as classification.

A geometric approach for image classification presented in [12] can be used to group image patches from different source images into smooth, stochastic and dominant orientation patches, based on the similarity of image geometric structure, such as edge and sharp line information. In addition, different ML methods have also been employed for the analysis of HS observations, such as SVMs and RFs, which have been considered for problems like object detection [13] and scene classification [14,15]. In the past 3–4 years, however, this field has been dominated by deep learning methods including CNNs [16]. Towards this direction, a recently released, eloquent survey-study concerning cutting-edge DL approaches in RS applications, can be found in [17].

An interesting work employing patch-based training of CNNs was conducted in [18], whereas a multi-scale CNN approach was proposed in [19] for HS image classification purposes. A pioneering work on the field was that in [20], with specific improvements concerning the model architecture being proposed in [21]. An early attempt of classifying 3D MS imagery was explored by [22], where features from each spectral band where interdependently extracted and fused together at a higher level. Extending the notion of convolution from 2 to 3 dimensions, in [23] is proposed a 3D-CNN for RS image classification, whereas, to address the challenges associated with the limited availability of training examples, works based on the notion of *Transfer Learning* were presented in [24,25]. Additionally, two quite informative reports on land-use and land-cover classification can be found in [16,26].

The above-mentioned cases involve the use of state-of-the-art ML classifiers applied on high-quality noise-free observations. However, due to several reasons, in real-world applications observations are affected by different types of noise, including noise due to the quantization of real-valued measurements, as well as missing observations. The probabilistic model that describes this type of observations was first introduced in [27], for the case of binary or 1-bit measurements. Under the assumption that the measurement matrix is low-rank, in [27] is proposed a convex program using maximum likelihood estimation and a nuclear norm to promote a low-rank solution. A similar approach is considered in [28], but with the max-norm in place of the nuclear norm. Finally, a constrained maximum likelihood estimation based on low-rank factorization is presented in [29].

An extension to multi-level observations is introduced in [30,31], using the same observation model and a constrained maximum likelihood estimator, either calculating the set of quantization bin boundaries from the observed data using an alternating optimization procedure or assuming that the quantization bin boundaries are known. In contrast to the previous observation model, which involves just one matrix, the observation model proposed in [32] involves a vector of K−1 underlying matrices for *K* level observations. Moreover, a maximum likelihood estimation from multi-level quantized observations based on matrix factorization is presented in [33].

Concerning the case of missing observations, the application of matrix and tensor completion of HS data is considered in [34] where the authors consider HS observation from mass spectral imaging and remote sensing and conclude that for cases of limited spectral resolution, exploiting the tensor structure of the data is advantageous while for large spectral resolution, matrix completion is preferable. The application of tensor completion for the recovery of missing values in imaging data is also considered in [35], where the authors proposed three optimization schemes for the recovery of missing values.

An interesting comparison of matrix and tensor-based methods for the recovery of missing environmental data takes place in [36], where the authors propose the exploitation of higher-order structural information, in favor of matrix-oriented techniques, when randomly distributed measurements are missing. In addition, in [37] the authors further examine the impact of completing higher-order structural information by matrix and tensor completion methods, both in the task of missing HS measurements’ reconstruction, as well as in that of subsequent classification.

Although numerous algorithms have been presented for the recovery of the real-valued entries of a matrix from its quantized measurements, no prior work has been conducted for the recovery of a tensor from multi-level quantized observations. However, in the case of binary measurements, a maximum likelihood estimator using a nuclear norm constraint on the different matricizations of the underlying tensor is proposed in [38]. A tensor recovery method from binary measurements is also presented in [39], which minimizes a loss function that incorporates a generalized linear model with the tensor nuclear norm as a regularization term, using an alternating direction method of multipliers-based algorithm to find the solution. Finally, a tensor-based method that uses the decomposition of the tensor in order to retain its structure, rather than matricization, is proposed in [40], in which a certain atomic M-norm as a convex proxy for rank is used to approximate low-rank tensors from binary measurements.

The concepts of matrix and tensor completion have also been considered for the efficient on-board compression of HS imagery. Matrix completion is employed for HS compression, targeting the on-board processing of observations in [41], where the authors propose a new method for the enhancement of the spatio-spectral resolution of hypercubes acquired by snapshot spectral imagers. In [42] the authors explore the potential of matrix and tensor completion as a compression mechanism with dramatically fewer computational requirements compared to more traditional transform coding schemes like JPEG2000. To fully exploit the spatio-spectral correlations of HS imagery, they consider the group non-overlap sampling scheme and adaptive rank threshold selection, and demonstrate that the proposed scheme offers comparable performance to 3D-JPEG2000 on AVIRIS imagery using less computational resources.

Trying to capitalize on the inherent high dimensionality of HS data, tensor decompositions have also been proven to be a valuable HS image compression tool. In this flavor, the ability to express a 3D tensor encoding both spatial and spectral measurements in more compact forms through tensor decompositions is explored in [43]. Tensor decomposition of HS image is also explored in [44], where the authors consider the CANDECOMP/PARAFAC approach in order to express the 3D HS cube as the sum of rank-1 tensors and exploit the sparsity of this representation for compression.

Finally, the impact of compression in the classification task of remote sensing images considered in the proposed scheme has also been examined with different compression and classification techniques. Specifically, the effects of JPEG2000 lossy image compression on the classification of WorldView-2 satellite images are explored in [45]. The authors consider the *k-Nearest Neighbors* (k-NN) and SVM classification methods, and experimentally demonstrate that even at high compression rates (30:1) there is a minimal impact on classification accuracy and in some cases, the results are even better than the original image classification. The same observation is established in [46] where the impact of lossy compression is investigated on *Principal Component Analysis* (PCA)-based spectral unmixing and SVM-based supervised classification using the CCSDS-ICS, TER and JPEG2000 compression schemes on AVIRIS data. However, these results indicate a strange behavior that cannot be completely trusted since very high compression ratios should have an adverse effect on classification accuracy.

Furthermore, the quantification of the impact of compression on the classification accuracy of satellite images is explored in [47], where a framework based on the *Multiple Kernel Learning* algorithm is employed for the prediction of how compression affects the quality of extracted features for classification. Specifically, the authors consider the JPEG2000 compression standard and the subsequent classification of each image into five classes (water, forest, bare land, vegetation and residential area) by two classifiers, namely a maximum likelihood and a pixel-level SVM classifier. However, the prediction performance of their method is evaluated on images with only three bands (near-infrared, red and green bands), from GF2, Landsat 8 and ZY-3.

In a similar vein, the impact of compression on synthetic PROBA-V images was investigated in [48], where the authors considered two compression schemes—the TER and the CCSDS blue book standard which evolved to the CCSDS-122 standard for lossless and near-lossless MS and HS image compression—and investigated the impact on land-cover estimation. The authors’ conclusion is that compression has a minimal impact on classification, which is more pronounced in regions with multiple boundaries between different land-cover classes. Similar observations were drawn for the case of Landsat 7 Enhanced Thematic Mapper Plus (ETM+) observation compression using JPEG2000, SPIHT and CCSDS 122.0-B-1 and its impact on two important vegetation features, namely *Normalized Difference Vegetation Index* and *Normalized Difference Water Index*, which are reported in [49]. Nevertheless, the above methods cannot handle cases of missing observations, due to communication failures or physical obstacles like clouds, in contrast to our method that can efficiently recover the missing values for the subsequent processing using a quantized tensor completion algorithm.

## 3. Problem Formulation and Proposed Method

In this section, we initially describe the scenarios under investigation for the classification task of quantized MS images. Subsequently, we provide some background concerning the reasoning of choosing CNNs to tackle the land-cover classification task, as well as the formulation of the problem at hand via application of two-dimensional filters on MS datacubes. Furthermore, basic mathematical details on how CNNs can be used for performing the estimation task are provided, accompanied by the proposed methodology for classifying MS images. In addition, the description of the measurement model, as well as the method for the recovery of the real-valued MS images from several quantized measurements via matricization of the tensor, are provided.

### 3.1. Quantized Multispectral Imagery Classification

Quantization is an integral part of data acquisition, especially for the RS scenario where full data transmission introduces considerable challenges in terms of data storage and data transfer. Therefore, an important study concerns the effect of quantization on the classification task.

**Figure 1:**
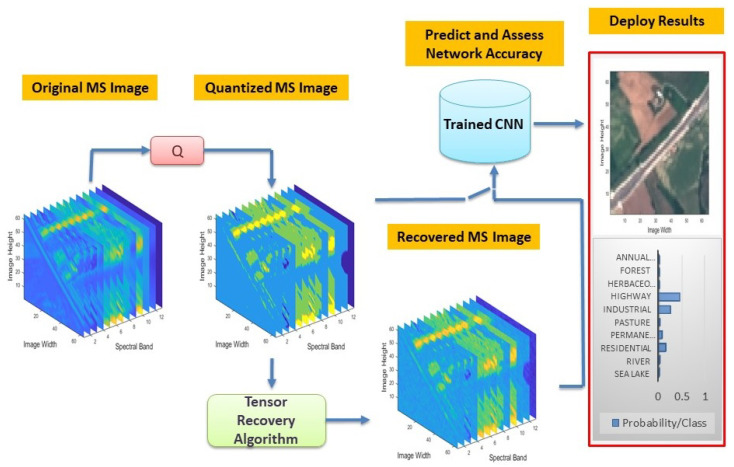
The scenarios under investigation for the classification task of compressed MS images.

The simplest approach for the classification task is to operate directly on the quantized—to a specific number of bits—MS images, using a trained CNN classifier, as illustrated in Figure 1. However, instead of classifying the quantized images, one can apply a tensor-based recovery algorithm, as the one introduced in this paper, for the reconstruction of the real-valued images. Experimental results on real satellite MS images demonstrate that directly processing using the quantized measurements, rather than recovering their real values, ends up with a significantly higher error-indicating the efficacy of the proposed method.

### 3.2. Deep Neural Networks for Land-Cover Classification

When applying DL techniques for predictive land-cover purposes, the charged statistical learning model can be naturally compared to that of human beings: learn by (training) examples. In this context, training data (e.g., RGB/MS/HS images) is used to derive a prediction concerning the class to which a specific image scene belongs to. In order for the model to have as high representational power as possible, this prediction ability should generalize well on new (unseen-test) data, on which there is no ground truth. To do so, models are trained by using a large set of labeled data and *Neural Network* (NN) architectures that learn features directly from the data without the need for manual feature extraction.

Putting things into a stricter mathematical perspective, predictive modeling through NNs can be described as the goal of approximating a (in general, non-linear) mapping function from input variables to output variables. In the case described in this paper, the input variables correspond to image pixel values, whereas the output variable is the (discrete) label value of the predicted class corresponding to the current piece of image content being considered. The reasoning behind such an expressive power (and the outstanding classification accuracy results obtained, consequently) of NNs is explained by their characterization as *universal approximators* [50,51]. That means that NNs can approximate arbitrarily well any given function, with the error of approximation being inversely proportional to the system’s order (i.e., the number of neurons). As a consequence, compensating for the non-linear optimization nature of the problem (hence the possibility of converging to local minima), NNs can achieve remarkable performance vis-à-vis conventional predictive modeling methods, and therefore are employed in the present experimental study.

#### 3.2.1. Spatial Feature Learning with Convolutional Neural Networks

CNNs constitute one of the most popular types of *Deep Neural Networks*. A CNN convolves learned features with input data, and uses 2D convolutional layers, making this architecture well suited to processing 2D data, such as images. A CNN can have tens or hundreds of layers, each one of whom learns to detect different features of an image. Filters are applied to each training image at different resolutions, and the output of each convolved image is used as the input to the next layer. The filters can start as very simple features, such as brightness and edges, and increase in complexity to features that uniquely define the object.

The proposed method fits within classification-flavored supervised learning, employing second-order CNNs for efficient spatial feature learning of the image content. Unlike conventional NNs, CNNs employ the notion of local receptive fields to effectively extract features from raw data. More specifically, each locally connected input subset of the input neuron is mapped to a single output neuron, a process which is performed in a stacked manner throughout convolutional layers, in order to capture as many representative features as possible. The connection between input and output neurons is performed via convolutions by means of trainable kernels, namely filters with specific filter coefficients. The number of such filters can be trimmed using pooling layers to avoid over-fitting issues.

Formally, the value of a convolved output neuron at position (k,l) in the j-th feature map of the i-th layer can be expressed as follows:

(1) $$y_{i,j}^{k,l}=f\left(\sum_{i=0}^{H-1}\sum_{j=0}^{W-1}w_{i,j}x_{\left(k+i)(l+j\right)}+b_{i,j}\right),$$

where f(.) is an activation function, $w_{i,j}$ stands for the value of the kernel connected to the current feature map at the position (i,j), $x_{\left(k+i)(l+j\right)}$ represents the value of the input neuron, $b_{i,j}$ is the bias of the computed feature map, and *H* and *W* are the height and width of the kernel respectively.

#### 3.2.2. Multispectral Image Prediction Modeling

Creating a network from scratch with the aforementioned procedure, translates to clear and total determination of the network configuration by the designer. This approach provides the most control over the network and can end up with impressive results, but, at the same time, requires an understanding of the structure of a neural network and the many options for layer types and configuration.

On the other hand, fruitful intuition can be gained by several popular pre-trained network architectures commonly used for image processing applications, such as AlexNet [52] and GoogleNet [53]. The majority of the pre-trained networks are trained on a subset of the ImageNet database (http://www.image-net.org/), which is used in the *ImageNet Large-Scale Visual Recognition Challenge* ( ILSVRC) [54]. These networks have been trained on more than a million images and can classify images into 1000 object categories, such as keyboard, coffee mug, pencil, and many animals. The reason for focusing on these architectures is that by learning a rich set of features they behave remarkably well in practice for a *similar* task to the problem at hand (RGB instead of MS image classification). Due to the different nature of the problem though, intuition can be gained by obtaining only their architecture and not their weights as well.

**Figure 2:**
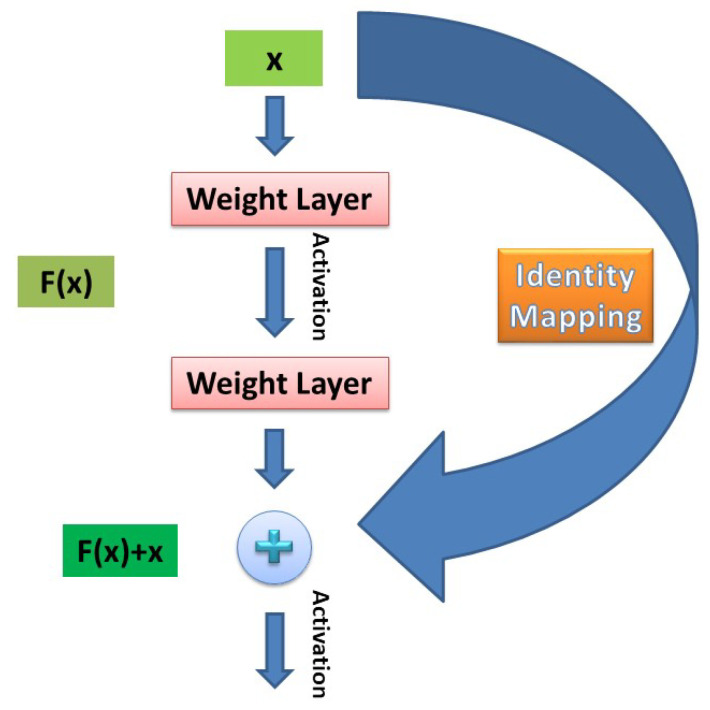
Residual learning framework with skip connections proposed in [55]. Instead of learning an unreferenced mapping (e.g., H(x)), the network learns a residual mapping (e.g., F(x)+x, with F(x)=H(x)−x). In this simple case, shortcut connections perform *identity* mapping, and subsequently add their output to the convolutional layers’ stack outputs.

Based on the aforementioned remark, the architecture of the CNN used throughout this paper is the one proposed in [55], inspired by an innovative work in image recognition. Therein, the authors build up on the framework of *Residual Learning* to argue about the reasoning of deeper architectures vis-à-vis swallow ones. To do so, each layer is charged not to learn unreferenced functions-mappings, but residual functions with reference to its inputs. Such a task is implemented by introducing *Shortcut Connections* (i.e., connections that skip 1 or more network layers), right after every stack of at least 2 convolutional layers, as shown in Figure 2.

From a theoretical point of view, our choice of using a specific version of residual networks as the appropriate model in the present study is based on the argument proven in [55], stating that deeper residual networks address more effectively the problem of *degradation* than their shallow counterparts. In other words, residual networks do not suffer from accuracy saturation and subsequent degradation as the depth of the network increases.

**Figure 3:**
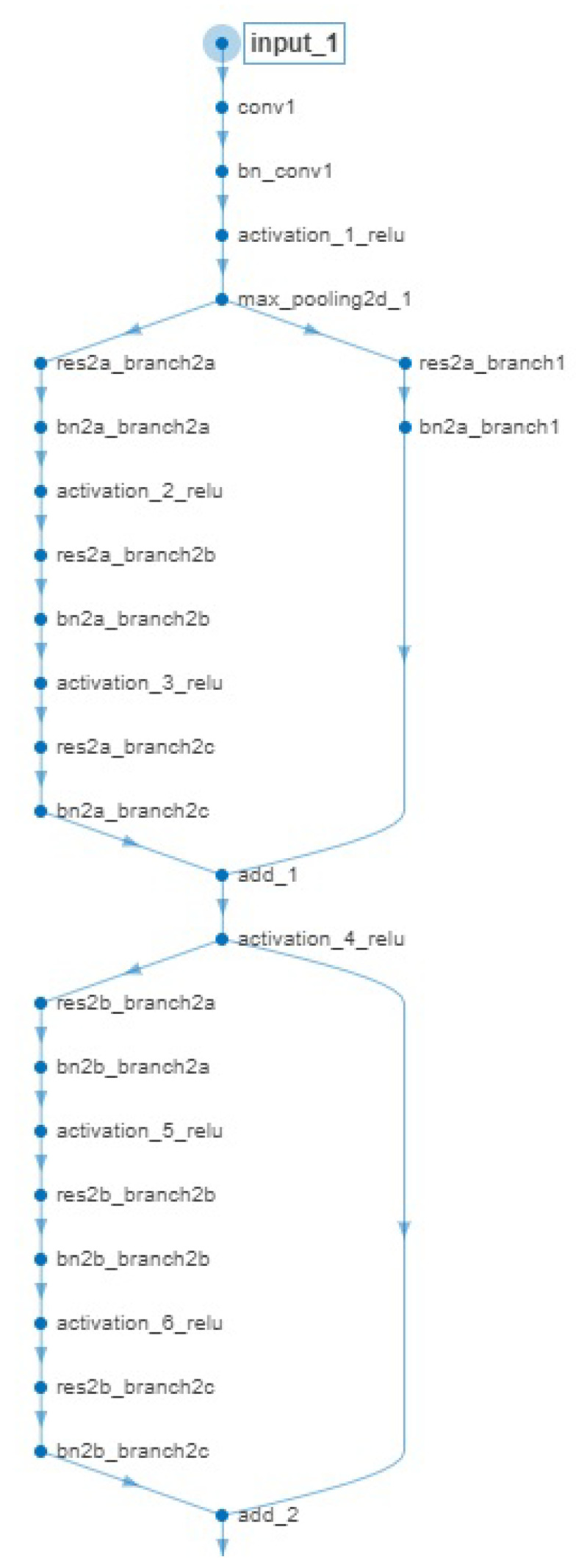
ResNet-50 layer graph. The employed network comprises several convolutional, activation, batch-normalization layers, connected via the notion of shortcut connections.

We chose the ResNet-50 model as the residual architecture. The network is 50 layers deep, comprising of approximately 25.6 million parameters (i.e., weights). As the whole ResNet-50 network architecture is quite extended, in order to meet space limitations, we depict in Figure 3 a small part of the network from which it becomes evident that layers can have inputs from multiple layers and outputs to multiple layers, as well.

The pattern “family of operations” described in Figure 3 is repeated several times, one right after the other, in the whole network architecture in order to formulate the final model. Although the layers comprising the network architecture can be considered to be conventional (i.e., conv-convolutional, bn_conv-batch normalization, activation, max-pooling), it is of interest to note the flow-split in multiple branches operating on par and adding up their processing results to be propagated to the next “family of operations”. We selected the ResNet-50 model over a shallower network of the same family (e.g., ResNet-18) because the deeper the architecture, the lower the classification error obtained [55].

Apart from the residual learning-based pre-trained models, several others have been proposed in the bibliography for addressing the ILSVRC and imaging tasks in general. When choosing which one to select for a specific task, one should take into consideration characteristics such as network speed, accuracy, and size (apart from task-specific issues). All of these play a crucial role in the selection (as long as a fast and highly accurate model is always desired), but usually there exists a trade-off between them due to “priority-and-resource” reasons. Our choice of ResNet-50 rested upon choosing a model with a good accuracy performance and a manageable computational burden.

In terms of the training process followed, as long as our intention is to classify MS images (instead of RGB ones) into significantly fewer than 1000 classes (for which ResNet-50 model is originally trained), we adopted the following steps:

1. Load the pre-trained ResNet-50 network, which has been trained for a task of similar flavor (i.e., RGB image classification) to that at hand (i.e., MS image classification).
2. Replace the classification layers (i.e., 1000 different classes in the ImageNet database) for the new MS image classification task.
3. Train the network on the available dataset for the MS image classification task.
4. Test accuracy of the trained network.

Regarding the optimization process followed during the training of the network, instead of the standard *Stochastic Gradient Descent* (SGD) optimizer, we employed Adagrad [56] with a learning rate of 0.001. The latter one was selected due to its adaptive selection of reducing the learning rate of parameters with high gradients and, conversely, increasing the learning rate of parameters which have small or infrequent updates. In order to recast the network’s outputs as probabilities, categorical cross-entropy was used as the loss function, as long as the cross-entropy can be interpreted as the log-likelihood function of the training samples.

### 3.3. Tensor Recovery from Quantized Measurements

In this section, we present our proposed method for the recovery of the real values of a MS image from its partial noisy and quantized measurements, in combination with a quantization and statistical model of our observations.

#### 3.3.1. Quantization and Statistical Model

Let $M\in R^{I_{1}\times \ldots \times I_{N}}$ be the *N*-th order tensor that models a high-dimensional signal, like a MS image, whereby the order of the tensor is defined as the number of its dimensions, also known as ways or modes. If $\Omega \subseteq \left\{1,\ldots ,I_{1}\right\}\times \cdots \times \left\{1,\ldots ,I_{N}\right\}$ is the index set of observed entries, $P_{\Omega }$ is a random sampling operator which keeps the entries in Ω and zeros out others, retaining only several entries from the tensor, i.e.,

(2) $$P_{\Omega }\left(M\right)=\left\{\begin{array}{cc} M_{i_{1}\ldots i_{N}} & \mathrm{if}\;(i_{1},\ldots ,i_{N})\in \Omega \\ 0 & \mathrm{otherwise} \end{array}\right..$$

The quantized and corrupted observations are related to the underlying tensor M via a probabilistic model, as follows. Given the tensor M and a twice differentiable function $f_{l}:R\to \left[0,1\right]$, with l∈{1,…,K},K≥2, we observe

(3) $$Y_{i_{1}\ldots i_{N}}=l\;\mathrm{with}\;\mathrm{probability}\;f_{l}\left(M_{i_{1}\ldots i_{N}}\right),\left(i_{1},\ldots ,i_{N}\right)\in \Omega ,$$

where $\sum_{l=1}^{K}f_{l}\left(M_{i_{1}\ldots i_{N}}\right)=1$. This model can be considered to be the *K*-level quantization of noisy measurements $M_{i_{1}\ldots i_{N}}+E_{i_{1}\ldots i_{N}}$, where $Y_{i_{1}\ldots i_{N}}$ is given by

(4) $$Y_{i_{1}\ldots i_{N}}=Q\left(M_{i_{1}\ldots i_{N}}+E_{i_{1}\ldots i_{N}}\right),\left(i_{1},\ldots ,i_{N}\right)\in \Omega .$$

The noise tensor E has i.i.d. entries with *Cumulative Distribution Function* (CDF) Φ(z), and the function Q:R→{1,…,K} corresponds to a uniform quantizer that maps a real number to a set of *K* ordered labels according to

(5) $$Q\left(x\right)=l\;\mathrm{if}\;w_{l-1}<x\leq w_{l},\;l\in \left\{1,\ldots ,K\right\},$$

where $\left\{w_{0},w_{1},\ldots ,w_{K}\right\}$ represents the set of quantization bin boundaries of all measurements, which satisfies $w_{0}\leq w_{1}\leq \cdots \leq w_{K}$. We will assume that the set of quantization bin boundaries is known a priori.

In terms of the likelihood of the observations $Y_{i_{1}\ldots i_{N}}$, the model in (4) can be written equivalently as

(6) $$f_{l}\left(M_{i_{1}\ldots i_{N}}\right)=P\left(Y_{i_{1}\ldots i_{N}}=l|M_{i_{1}\ldots i_{N}}\right)=\Phi \left(U_{i_{1}\ldots i_{N}}-M_{i_{1}\ldots i_{N}}\right)-\Phi \left(L_{i_{1}\ldots i_{N}}-M_{i_{1}\ldots i_{N}}\right),$$

where the $I_{1}\times \ldots \times I_{N}$ tensors U and L contain the upper and lower bin boundaries corresponding to the measurements $Y_{i_{1}\ldots i_{N}}$.

We consider two common choices for E and its associated CDFs:

- The logistic model (logistic noise), which is common in statistics, with $E_{i_{1}\ldots i_{N}}$ i.i.d. according to the logistic distribution with zero mean and unit scale, and $\Phi_{\log}\left(x\right)=\frac{1}{1+e^{-x}}$.
- The probit model (standard normal noise) with $E_{i_{1}\ldots i_{N}}$ i.i.d. according to the standard normal distribution N(0,1), and $\Phi_{\mathrm{pro}}\left(x\right)=\int_{-\infty }^{x}N\left(s|0,1\right)\;ds.$

The proposed algorithm can be formulated for both noise models.

#### 3.3.2. Quantized Tensor Recovery

A common framework for tensor computations is to turn the tensor into a matrix, as it is easier to handle matrices instead of third- or higher-order tensors. The process of reordering its elements into a matrix is called matricization or unfolding of the tensor. Specifically, given the measurement tensor $Y\in R^{I_{1}\times \ldots \times I_{N}}$, its mode-*n* matricization is denoted as unfold${}_{n}\left(Y\right)=Y_{\left(n\right)}\in R^{I_{n}\times \prod_{j\neq n}I_{j}}$, and corresponds to a matrix with columns being the vectors obtained by fixing all indices of Y except the *n*-th index [57]. The reverse process, which is the folding of a matrix to a tensor along mode-*n*, is denoted as $\;{\mathrm{fold}}_{n}\left(Y_{\left(n\right)}\right)=Y$.

Fortunately, the high-dimensional data often lie in a low-dimensional subspace by removing potential noise, which can be translated into the low-rank assumption on the recovered tensor. However, there is no straightforward algorithm to determine the rank of a specific given tensor, as it is an NP-hard problem [58]. Therefore, we can apply a nuclear norm constraint on each unfolding of the tensor, which is a convex relaxation of the low-rank constraint [59].

More, formally, in order to recover the real values of the low-rank tensor $M\in R^{I_{1}\times \ldots \times I_{N}}$ from partial quantized and possibly corrupted observations, we unfold the measurement tensor $Y\in R^{I_{1}\times \ldots \times I_{N}}$ into *N* matrices and for each of them, we apply the following algorithm. Then, the estimated tensors of each unfolding, $Z_{n}=\;{\mathrm{fold}}_{n}\left(Z_{\left(n\right)}\right),n=1,\ldots ,N$, are produced by folding each of the recovered matrices $Z_{\left(n\right)}$, and synthesize the recovered tensor such that:

(7) $$M\approx \sum_{n=1}^{N}a_{n}\cdot Z_{n}$$

where $a_{n},\;n=1,\ldots ,N,$ are weights, which depend on the fitting error, and satisfy $\sum_{n}a_{n}=1.$

The proposed algorithm can be regarded as an extension of the quantized matrix recovery [30] to the quantized tensor recovery. In particular, in order to recover the low-rank mode-*n* matricization $M_{\left(n\right)}$ from a subset of its quantized measurements, we use a constrained maximum likelihood approach. We assume Y is related to M via the probabilistic model given in (3)–(6). Then, the negative log-likelihood function is given by

(8) $$F_{Y_{\left(n\right)}}\left(X\right)=-\sum_{j,k:\left(j,k\right)\in \Omega_{n}}\left[\sum_{l=1}^{K}\log\left(f_{l}\left(X_{jk}\right)\right)1_{\left[{Y_{\left(n\right)}}_{jk}=l\right]}\right],$$

where $\Omega_{n}$ is the index set of observed entries of $M_{\left(n\right)}$. The function $F_{Y_{\left(n\right)}}$ is convex in X when the function $f_{l}$ is log-concave in $X_{jk}$. Both choices for the function $f_{l}$ are log-concave. Therefore, we seek to solve the following constrained optimization problem:

(9) $$\begin{array}{cc} \mathrm{minimize}{\;}_{M_{\left(n\right)}} & -\sum_{j,k:\left(j,k\right)\in \Omega_{n}}\log P\left({Y_{\left(n\right)}}_{jk}|{M_{\left(n\right)}}_{jk}\right)\\ \mathrm{subject}\;\mathrm{to} & ∥M_{\left(n\right)}{∥}_{*}\leq \lambda . \end{array}$$

The nuclear norm constraint $∥M_{\left(n\right)}{∥}_{*}\leq \lambda$ promotes low-rankness of $M_{\left(n\right)}$ [59] and the parameter λ>0 is used to control its rank.

Since the gradient of the negative log-likelihood of the inverse logit and probit link functions are convex in $M_{\left(n\right)}$ when keeping the quantization bin boundaries $w_{0},w_{1},\ldots ,w_{K}$ fixed, the optimization problem in (9) can be solved efficiently. Starting with an initialization of the estimated matrix $Z_{\left(n\right)}$ as a random matrix $Z_{\left(n\right)}^{1}$ with entries between the corresponding quantization bin boundaries, the algorithm performs two steps at each iteration l=1,2,…. Both steps are repeated until a maximum number of iterations $l_{\max}$ is reached or the change in $Z_{\left(n\right)}$ between consecutive iterations is below a given threshold.

The first step aims at reducing the objective function $F_{Y_{\left(n\right)}}\left(Z_{\left(n\right)}\right)$ of (9) and is given by

(10) $${\hat{Z}}_{\left(n\right)}^{l+1}\leftarrow Z_{\left(n\right)}^{l}-\frac{s_{l}}{\sqrt{l}}\cdot \nabla F_{Y_{\left(n\right)}},$$

where $s_{l}$ is the step-size at iteration *l*. For simplicity, we use a constant step-size $s_{l}=\frac{1}{L}$, where *L* is the Lipschitz constant, which is given by $L_{\log}=\frac{1}{4}$ for the logistic model and $L_{\mathrm{pro}}=1$ for the probit model. The gradient of the objective function $F_{Y_{\left(n\right)}}\left(Z_{\left(n\right)}\right)$ of each measurement, with respect to $Z_{\left(n\right)},$ is given by

(11) $${\left[\nabla F_{Y_{\left(n\right)}}\right]}_{jk}=\left\{\begin{array}{cc} \frac{\Phi^{{}^{\prime }}\left({L_{\left(n\right)}}_{jk}-{Z_{\left(n\right)}}_{jk}\right)-\Phi^{{}^{\prime }}\left({U_{\left(n\right)}}_{jk}-{Z_{\left(n\right)}}_{jk}\right)}{\Phi \left({U_{\left(n\right)}}_{jk}-{Z_{\left(n\right)}}_{jk}\right)-\Phi \left({L_{\left(n\right)}}_{jk}-{Z_{\left(n\right)}}_{jk}\right)} & \mathrm{if}\;\left(j,k\right)\in \Omega_{n}\\ 0 & \mathrm{otherwise} \end{array}\right.,$$

where $L_{\left(n\right)}$ and $U_{\left(n\right)}$ are the mode-*n* matricizations of L and U and contain the lower and the upper bin boundaries of the observations ${Y_{\left(n\right)}}_{jk}$ respectively, i.e., $L_{\left(n\right)}={\mathrm{unfold}}_{n}\left(L\right)$ and $U_{\left(n\right)}={\mathrm{unfold}}_{n}\left(U\right).$ The derivative of the function Φ(x) can be calculated as $\Phi_{\log}^{{}^{\prime }}\left(x\right)=\frac{1}{2+e^{-x}+e^{x}}$ and $\Phi_{\mathrm{pro}}^{{}^{\prime }}\left(x\right)=N\left(x|0,1\right)$ for each model.

The second step aims to impose low-rankness on $Z_{\left(n\right)}$ to make the solution satisfy the constraint $∥Z_{\left(n\right)}{∥}_{*}\leq \lambda .$ To achieve this, we take the SVD, $\tilde{U}S{\tilde{V}}^{T}$, of the matrix ${\hat{Z}}_{\left(n\right)}^{l+1}$ of the previous step, and we hold some of its singular values s=diag(S), depending on the parameter λ. In our experiments, we keep 97% of the information of the singular values.

#### 3.3.3. Dynamic Weights

The weights $a_{1},\ldots ,a_{N}$ in (7) can be fixed as $a_{n}=\frac{1}{N},\;n=1,\ldots ,N$, indicating that the recovered tensor of each unfolding equally contributes to the recovery. However, in some cases, the recovery in one unfolding may be better than others. Therefore, instead of fixed weights, we use dynamic weights which depend on the fitting error

(12) $${\mathrm{fit}}_{n}\left(Z_{\left(n\right)}\right)={∥P_{\Omega }\left({\mathrm{fold}}_{n}\left(Z_{\left(n\right)}\right)-Y\right)∥}_{F},$$

where ${∥X∥}_{F}=\sqrt{<X,X>}$ is the Frobenius norm of X (with $<X,Y>=\sum_{i_{1}=1}^{I_{1}}\ldots \sum_{i_{N}=1}^{I_{N}}x_{i_{1}\ldots i_{N}}y_{i_{1}\ldots i_{N}}$ denoted to be the inner product of $X,Y\in R^{I_{1}\times \ldots \times I_{N}}$). The smaller ${\mathrm{fit}}_{n}\left(Z_{\left(n\right)}\right)$ is, the larger $a_{n}$ should be. Specifically, we set

(13) $$a_{n}=\frac{{\left[{\mathrm{fit}}_{n}\left(Z_{\left(n\right)}\right)\right]}^{-1}}{\sum_{i=1}^{N}{\left[{\mathrm{fit}}_{i}\left(Z_{\left(i\right)}\right)\right]}^{-1}},\;n=1,\ldots ,N.$$

As demonstrated below, the dynamic weights $a_{n}$ can improve the recovery quality of the recovered tensor.

## 4. Experimental Evaluation

In this section, the dataset on which upcoming experiments are performed is described, as well as the experimental setup followed. Results of the adopted approach under different experimental scenarios, using several configurations in order to quantify the effects of various parameters in the whole MS image classification pipeline, are presented and discussed, to illustrate the performance of the methodologies introduced in this paper.

### 4.1. Dataset Description

In designing DL solutions and training ML models, the definition of a suitable training and test set is a critical task. Bearing into mind that to achieve decent classification performance, the number of samples comprising the dataset must be sufficiently large, most current land-use and land-cover classification datasets did not meet these expectations. Furthermore, the “distribution” of samples (i.e., images) inside the dataset should be as close to uniform as possible (in order to avoid class imbalance problems), with each class consisting of several thousands of samples to be fed to the NN model.

Fortunately, quite recently, a large MS image dataset (containing RGB images as well) meeting the above requirements was released [60,61], where Sentinel 2A MS image data are employed to address land-use and land-cover classification tasks. The dataset comprises image patches measuring 64×64 pixels, each one of them corresponding to spatial resolution of 10 meters/pixel, across 13 different spectral bands (in the 443 nm–2190 nm wavelength range). In total, several thousands of sample satellite images are gathered, across 34 different European countries (hence, the EUROSAT dataset name), ending up with 10 different classes.

**Figure 4:**
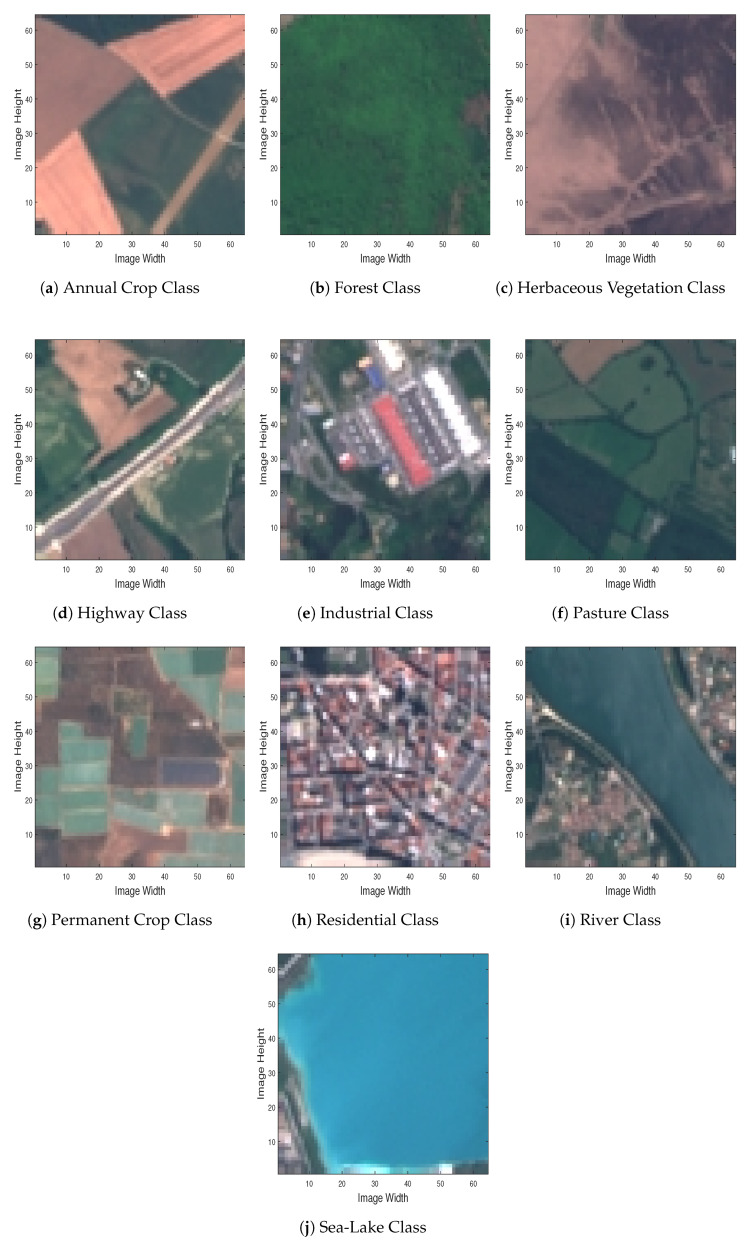
Sample RGB images of all different classes included in the EUROSAT dataset.

**Figure 5:**
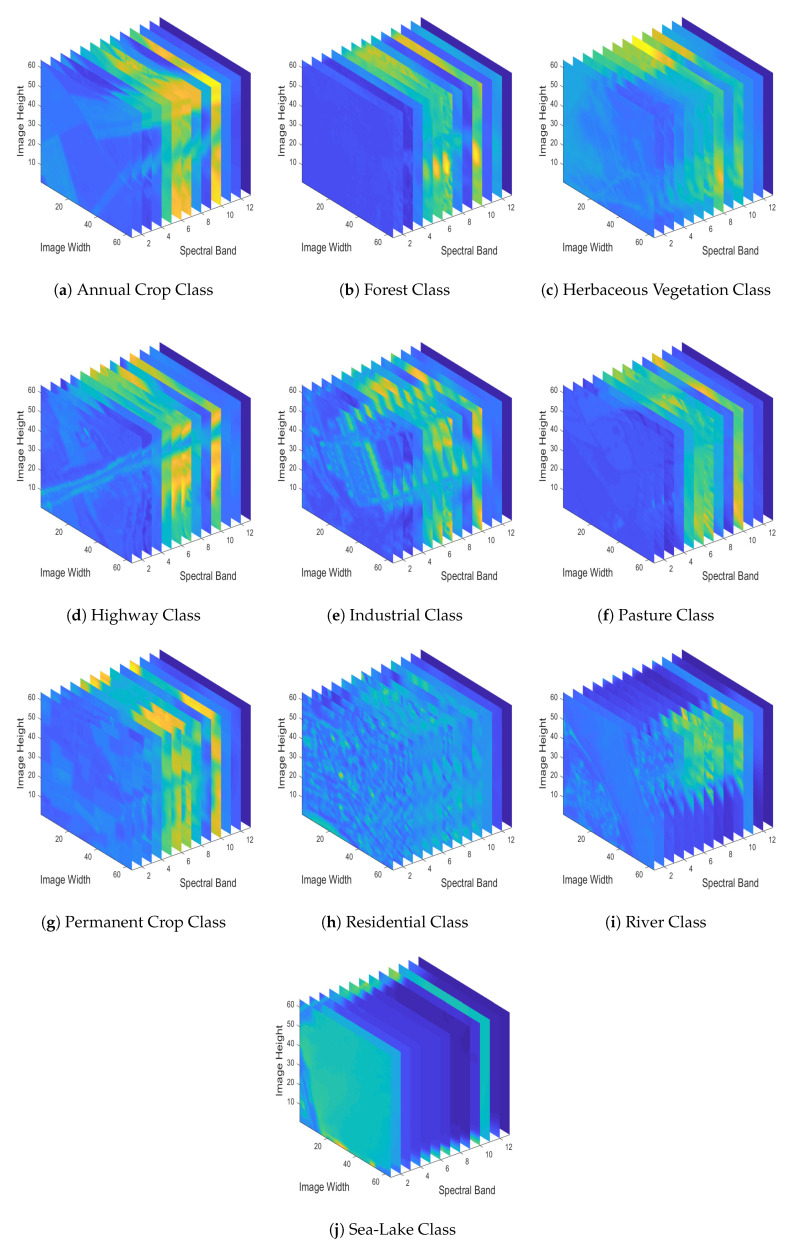
MS datacubes of sample images of all different classes included in the EUROSAT dataset.

To have a better sense of the EUROSAT dataset, in Figure 4 we depict RGB sample images for each one of the dataset’s different classes, by employing spectral bands 04 (Red-665 nm), 03 (Green-560 nm) and 02 (Blue-490 nm). Since in this study we deal with a MS image classification task, in Figure 5 we demonstrate the various spectral bands (MS datacubes) of the sample images depicted in Figure 4.

### 4.2. Experimental Setup

Since the classification task lies in the heart of the present study, two non-overlapping training-test sets had to be defined a priori. In order to avoid class imbalance problems, we employed 20,000 MS sample images from the EUROSAT dataset assuming that each class consists of 2000 samples. With this setup, we split each class into 90%/5% training/test sets (i.e., 1800/100 training/test examples per class, and 18,000/1000 training/test examples in total, respectively), while at the same time another 5% of each class was used for validation purposes during the training process of the network. Training was performed using a constant number of up to 100 epochs with the intention of observing how the prediction accuracy changes by increasing the number of epochs the network is trained for.

To make more clear the above data distribution among the training, validation, and test sets, we summarize in Table 1 the aforementioned splits for each of the employed classes as well as in total.

To quantify the performance of the proposed system from a ML perspective, we report *Classification Accuracy* and *Classification Loss* metrics, accompanied by the required *Computational Time* as well as the respective *Confusion Matrix*. Furthermore, *Precision* and *Recall* metrics are also computed. However, in order to examine the effect of the quantization on the classification task, we quantized the MS images of our dataset to 2, 4, 6, 8 and 10 bits, as the original MS images use 12 bits per pixel. Then, we compared the classification performance given the original, the quantized and the recovered images via the proposed recovery algorithm. Moreover, we examined the recovery of the real values of the images given the quantized measurements.

The reconstruction quality is evaluated by computing the *Peak-Signal-to-Noise-Ratio* (PSNR) between the original and the estimated MS images. Higher PSNR values correspond to a better-quality recovered image. Specifically, PSNR is computed using the expression

(14) $$PSNR=10\cdot \log_{10}\left(\frac{R^{2}}{MSE}\right),$$

where *R* is the maximum value of the input image and MSE is the *Mean Square Error*, i.e., the average of the squares of the differences between the original and the estimated signal.

Since the training of a DL model is a computational expensive procedure, the employed CNN architectures were developed in Python programming platforms-exploiting the TensorFlow (https://www.tensorflow.org/) and Keras (https://keras.io/) libraries. There are two reasons behind such a choice: First, TensorFlow is an open-source ML framework, which when used as the back-end engine for the higher-level DL-specific Keras library, provides support and updates on most state-of-the-art DL models and algorithms. Secondly, and most importantly, both libraries can perform calculations on a GPU, dramatically decreasing the computational time of the training process. In our experiments, we used NVIDIA’s GPU model, Quadro P4000.

On the other hand, since the compression algorithms described earlier do not need a GPU usage for their computations, they were implemented in MATLAB R2019a version.

### 4.3. Effect of the Training Set Size on the Classification Performance

In a first set of experiments, our objective is to assess the performance of the employed CNN relative to the size of the training set. A random selection of up to 200 training examples per class was initially considered, a number which was augmented by 200 at each step until containing all available training examples of each class (i.e., 2000 per class). The number of test examples was 100 per class (i.e., 1000 in total), as mentioned earlier.

**Figure 6:**
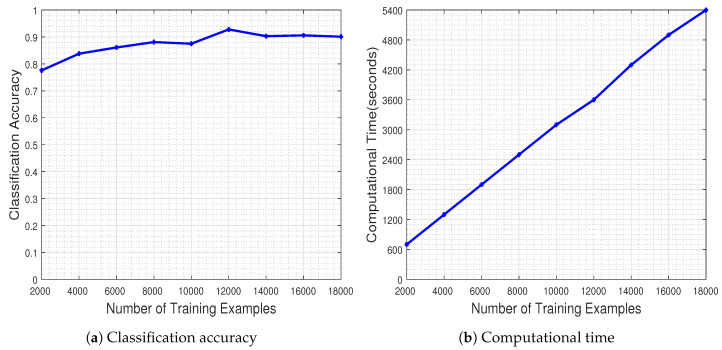
Classification accuracy and respective computational time regarding the number of training examples. The more training examples are used, the better the classification accuracy, translating to a more computational thirsty network.y

Figure 6 illustrates the performance of the CNN measured as a function of the number of the training examples. The results in Figure 6a demonstrate that increasing the number of training examples has a positive effect on the generalization capacity of the CNN, as dictated by the theoretical underpinnings. The network has a stable performance when trained with at least 8000 training examples. It should be noted that the CNN classifier achieves the best performance when trained with about 1200 image samples per class (i.e., 12,000 image samples in total), ending up with a classification accuracy equal to 92.80%.

Naturally, the improved classification performance of the CNN model does not come for free, as demonstrated by the execution time plots in Figure 6b. However, one should highlight the fact that on the one hand the time increases by a factor of 600 s as the number of training samples is doubled, while on the other hand the time needed for achieving the best performance at 12,000 training examples, namely 3600 s (i.e., 1 h), is far from being characterized as prohibitive considering typical DL experimental regimes. Overall, the results clearly demonstrate the merits and learning potential of the employed CNN modeling.

**Figure 7:**
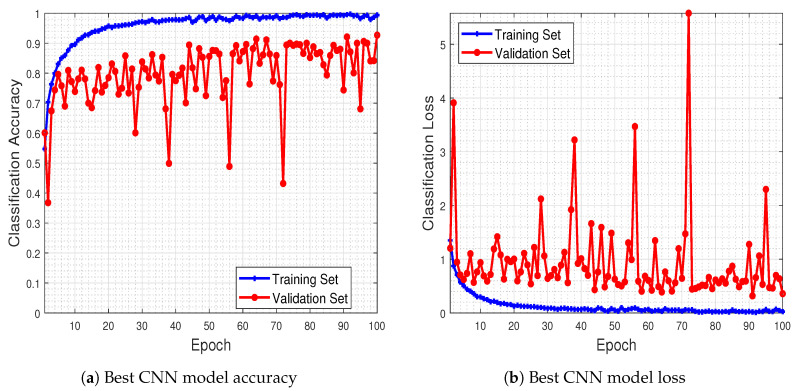
Best CNN model’s classification accuracy and loss regarding training epochs. As the number of epochs increases, so does the performance of the CNN model, both in terms of classification accuracy as well as in classification loss.

To have a better sense of the network’s behavior during the training process, in Figure 7 we provide the best model accuracy as well as its loss (i.e., for 12,000 training examples), for the training and validation sets, with respect to the number of epochs up to which the network was trained.

The results of this experiment show that the trained CNN model improves its performance in terms of classification accuracy, both in the training as well as in the validation set, as the number of epochs increases. Furthermore, the classification loss in the validation set keeps track, in general, towards that of the training set, showing that the model is capable of generalizing well during the test phase (which is indeed the case, as mentioned earlier in Figure 6, with the 92.80% classification accuracy in the test set).

**Figure 8:**
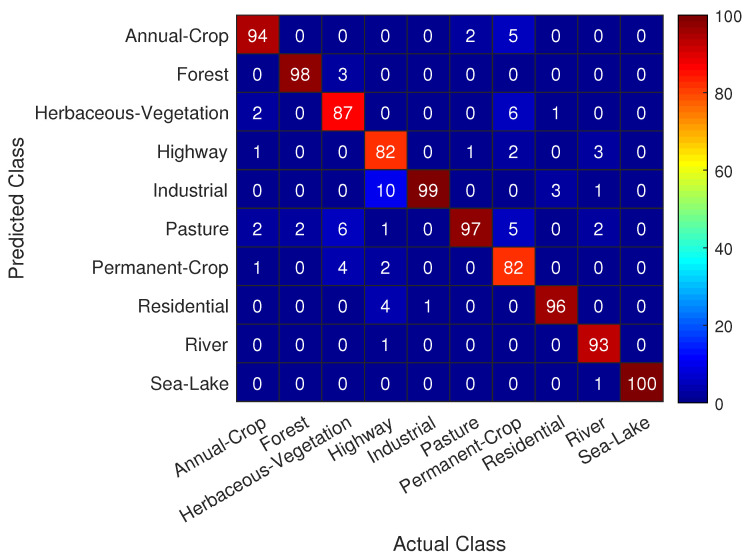
CNN model confusion matrix. For most classes in the test set, the trained CNN model correctly predicts the actual class with a success rate of over 90%.

Useful information concerning the classification task at hand can be extracted from the confusion matrix shown in Figure 8, where we see that the trained CNN achieves quite remarkable performance in predicting correctly nearly all actual classes in the test set with a success rate of over 90%. A special notice should be given to the classes that appear to be more challenging for correct prediction, namely:

- Class *Highway* is most frequently mistaken with classes *Industrial* and *Residential*
- Class *Permanent Crop* is most frequently mistaken with classes *Herbaceous Vegetation*, *Annual Crop* and *Pasture*

Even for these difficult to classify classes in Figure 4, the trained CNN model achieves a success rate of over 80%, indicating the efficacy of the proposed approach.

### 4.4. Effect of the Compression Ratio on the Recovery

As the quantization process can be used for compression purposes, we examined the efficacy of our method for different compression ratios on the MS images of each class, with the results presented in Table 2. Specifically, in order to compress the data, a lossless coding algorithm, namely Huffman coding, was employed on the quantized MS images, using a dictionary obtained from the images of the training set. The corresponding *Compression Ratio* is calculated as the fraction of the number of bits of the uncompressed image over the number of bits of the compressed one, i.e.,

(15) $$\mathrm{Compression}\;\mathrm{Ratio}=\frac{\mathrm{Bits}\;\mathrm{of}\;\mathrm{uncompressed}\;\mathrm{image}}{\mathrm{Bits}\;\mathrm{of}\;\mathrm{compressed}\;\mathrm{image}}.$$

As expected, the smaller the compression ratio, the better the performance of our algorithm. However, efficient recovery with a high compression ratio can still be achieved by our technique.

### 4.5. Effect of the Number of Quantization Bits on the Recovery

**Figure 9:**
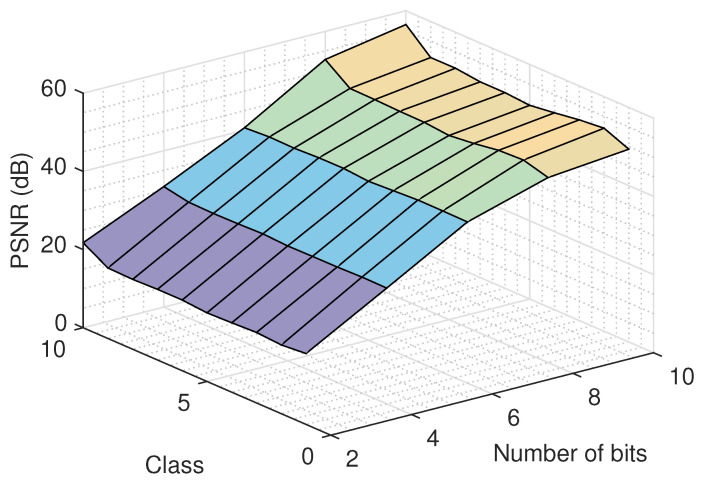
Recovery error as a function of the number of quantization bits and for different classes, using the logistic model.

An important issue that must be examined deals with the effect of the number of quantization bits. To study this, we run the proposed recovery algorithm for several quantization bit values and for various classes, using the logistic model, and we report the recovery error in Figure 9. As it was expected, a bigger number of quantization bits results to a better quality for the recovered images. Similar performance is observed for all classes. Moreover, the recovery improvement in the large number of bits region (8 and 10) is smaller compared to the improvement achieved in the small number of bits region (2 and 4), as more information is retained using more bits for the representation of the signal.

**Figure 10:**
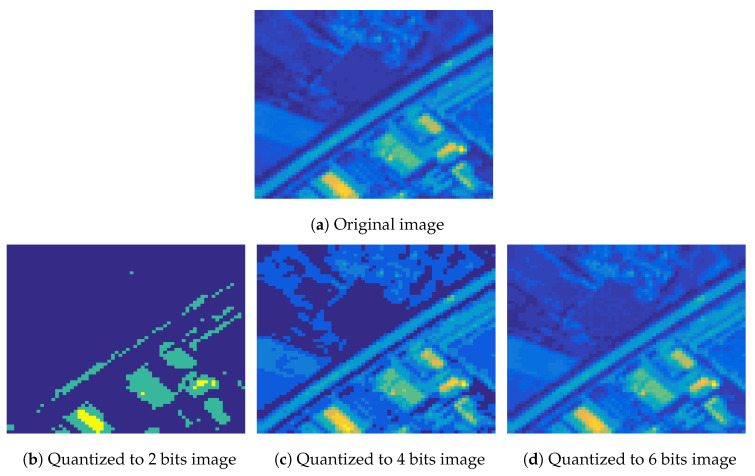

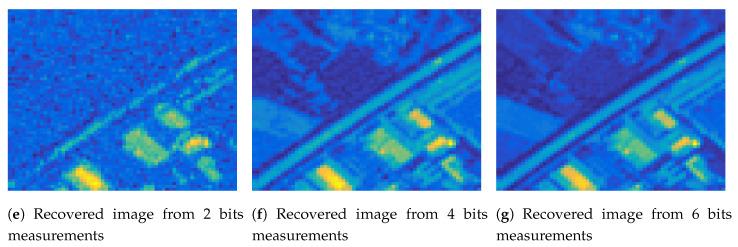
The second spectral band of a MS image from the Highway class, and the corresponding quantized to 2, 4 and 6 bits images, as well the recovered images for each case, using the logistic model.

The above observation can be visually verified in Figure 10 that depicts the second spectral band of a MS image from the *Highway* class and the corresponding quantized images using 2, 4 and 6 bits, as well the recovered images for each case, using the logistic model. As we observe, the quality of the recovered images from quantized measurements using 4 and 6 bits is better than the one using 2 bits. In addition, we can observe that using fewer bits, more information of the image is lost, which can be recovered using the proposed algorithm.

Please note that the recovery error for each class and a specific number of bits is calculated as the mean value of the error on the test images for all the experiments. In addition, we observed from our experiments that the performance of the method for the two noise models used was similar, so we kept the logistic model for the following experiments.

### 4.6. Effect of the Tensor Unfolding and Dynamic Weights on the Recovery

**Figure 11:**
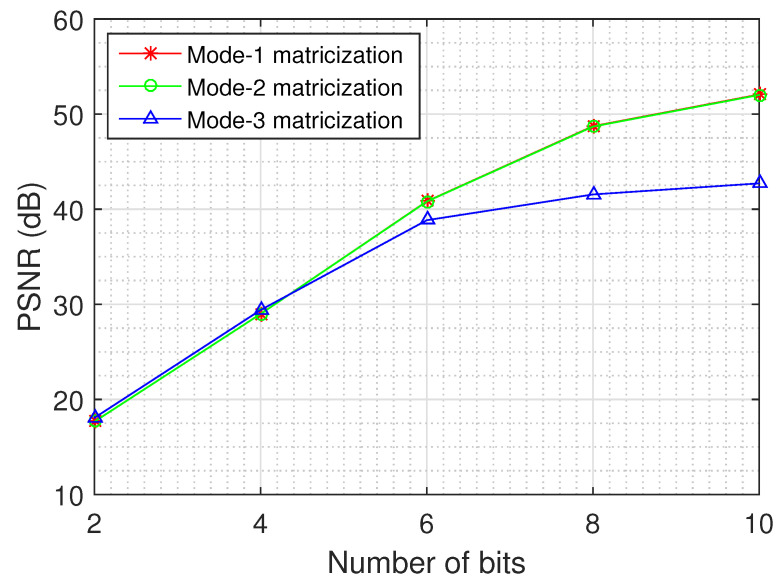
Recovery error as a function of the number of quantization bits on different unfoldings of the tensor, using the logistic model and the MS images of the Annual Crop class that indicate the impact of the dynamic weights on the recovery.

An important issue relates to the quality of the recovery for each unfolding of the tensor data and the impact of the dynamic weights on the recovery. Figure 11 depicts the recovery error as a function of the number of quantization bits for the different unfoldings of the tensor, using the logistic model and the MS images of the Annual Crop class.

The results demonstrate that the dynamic weights improve the quality of the recovered tensor, as the mode-1 and mode-2 matricizations exhibit a better performance than the mode-3 matricization, especially for large values of quantization bits (8 and 10). In addition, we can deduce from the results that the recovery performance becomes better when the dimensions of the unfolding matrix are more balanced, as the mode-1 and mode-2 matricizations of each MS image have dimensions 64×832, while the mode-3 matricization has dimensions 13×4096.

### 4.7. Effect of the Quantization on the Classification Performance

In this set of experiments, we investigate the detrimental effect that the quantization process has in the classification performance of a system. For that purpose, the trained CNN model is evaluated to a test set that has been previously quantized to a specific number of bits, in order to obtain intuition about the system response when faced with corrupted image samples.

**Figure 12:**
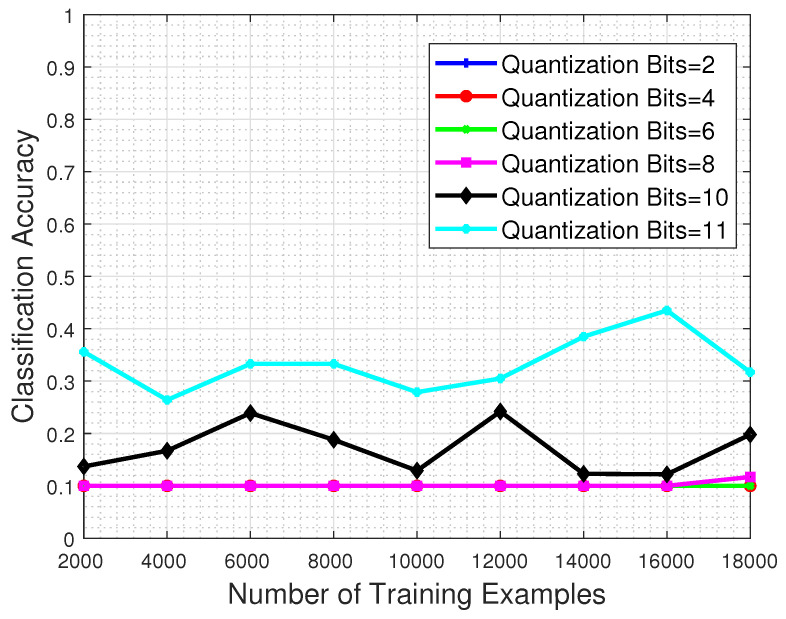
Classification accuracy regarding the number of training examples, for several quantization levels. The classification strength of the system clearly suffers even when the quantization process is performed to only 1 bit less than the nominal case.

Figure 12 shows the performance with respect to the number of training examples and for different quantization bit values imposed on the test set image samples. The results clearly demonstrate that whatever the quantization level, the classification performance clearly suffers even if the system is trained with the maximum permitted number of training examples. Even in the favorable case of 11-bit test images (recall that the nominal bits corresponding to each MS image are 12) the network’s classification performance barely reaches 40%.

### 4.8. Effect of the Quantization and the Recovery on the Classification Performance

To explore the ability of our CNN model to classify compression-processed MS images, we further investigate the enhancing effect that our proposed recovery process—of previously quantized MS images—has in the classification performance of a system. Towards that goal, the trained CNN model is evaluated using a recovered by our approach test set, which was previously quantized to a specific number of bits.

**Figure 13:**
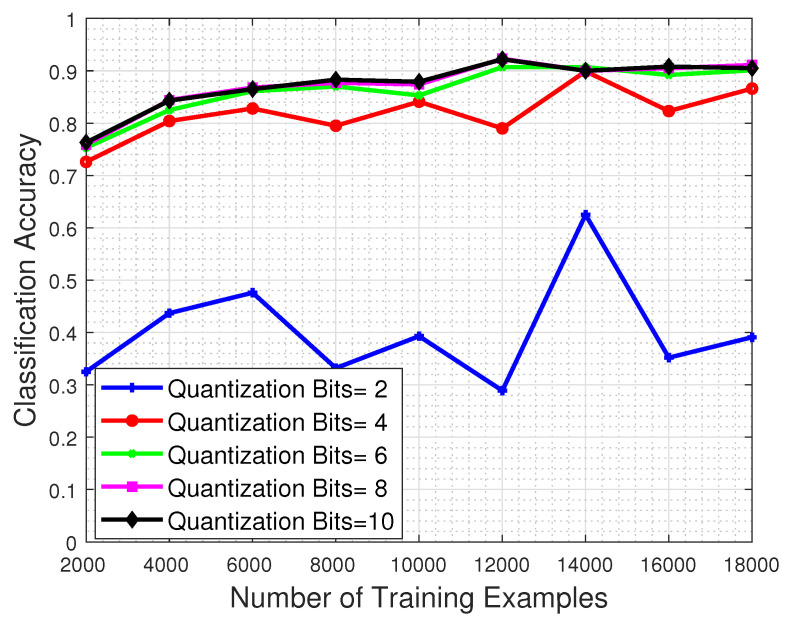
Classification accuracy regarding the number of training examples, for various levels of quantized images subsequently recovered using the proposed method. Our recovery approach clearly improves classification performance even when operating with images quantized with as few as 4 bits.

In Figure 13 we show the system’s performance as a function of the number of training examples, and for test images quantized to 2, 4, 6, 8, and 10 bits and subsequently recovered using our proposed method. The results clearly show our recovery approach manages to boost the classification accuracy of the system, even when operating with recovered images that were previously quantized using as few as 4 bits.

### 4.9. Joint Effects of the Quantization and the Recovery on the Classification Performance

In this set of experiments we make a general comparison of the aforementioned quantization and recovery processes, in order to quantify their effects in the MS image classification task, with respect to the “original” case where none of them is present in the classification pipeline.

**Figure 14:**
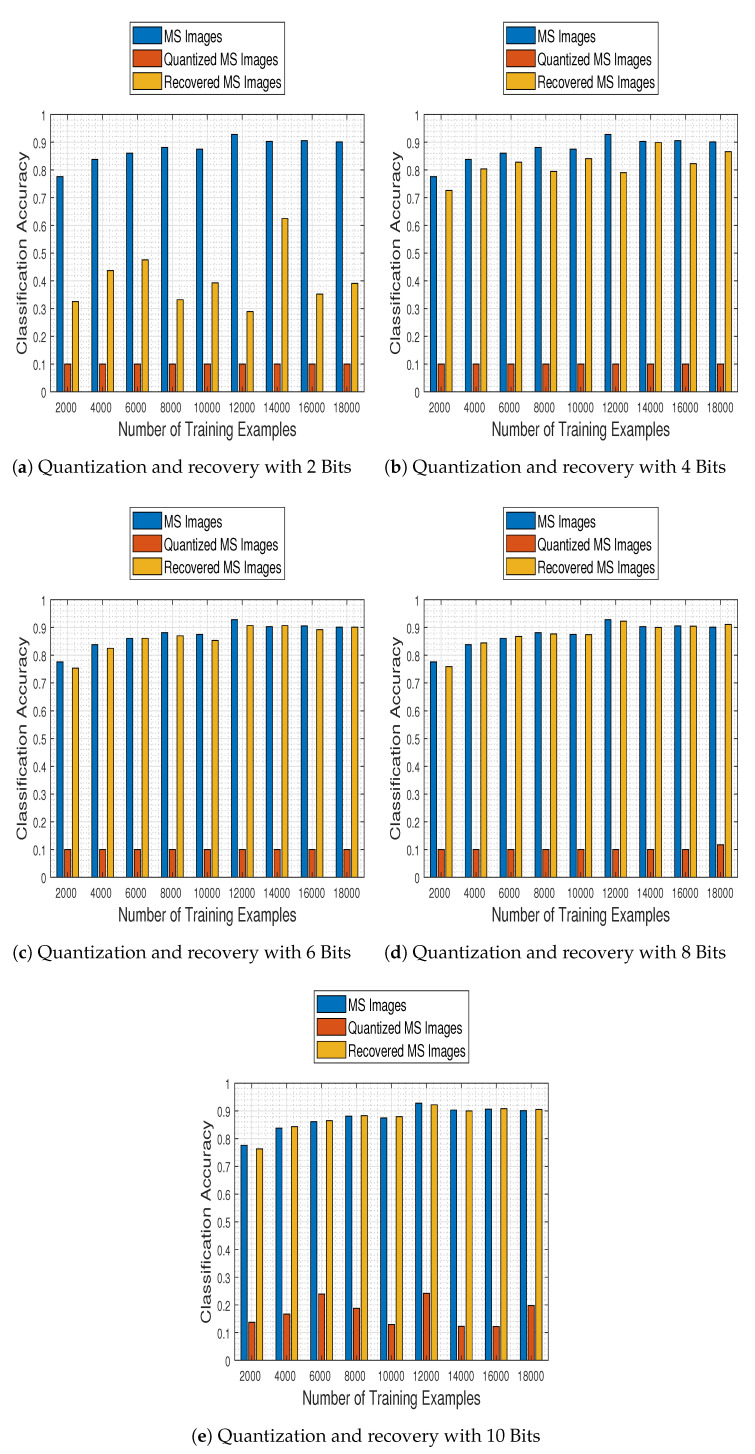
General comparison of the quantization and the recovery processes on the classification performance.

Figure 14 contains classification accuracy plot bars for the employed CNN trained with 2000:2000:18,000 samples on original test MS images, their quantized versions (using 2, 4, 6, 8, and 10 bits) and their recovered by our approach estimates. The results make clear that the proposed recovery method not only remedies the effects of quantization, but most interestingly it empowers the CNN model to exhibit a classification performance on par to that obtained when working on the original MS images, nearly from a compression level when the images are quantized to half the bits of their nominal available ones (6 bits).

### 4.10. Effect of Missing Values on the Recovery

In many cases, noise or other issues in the transmission and acquisition of a signal, lead to unobserved or lost measurements. One such case could be caused by the appearance of clouds in the acquired images. Hence, the issue we address concerns missing pixel patches, within all image spectral bands, a problem more challenging than the incoherent condition usually assumed in tensor completion, where the missing elements must be randomly distributed in the tensor.

To examine the effect of missing values in the data, we randomly selected patches of each test image, imposing missing elements across all spectral bands. In particular, we considered the following sampling scenarios:

1. 10 patches of size 3 × 3 × 13 pixels
2. 20 patches of size 3 × 3 × 13 pixels
3. 10 patches of size 7 × 7 × 13 pixels
4. 20 patches of size 7 × 7 × 13 pixels

**Figure 15:**
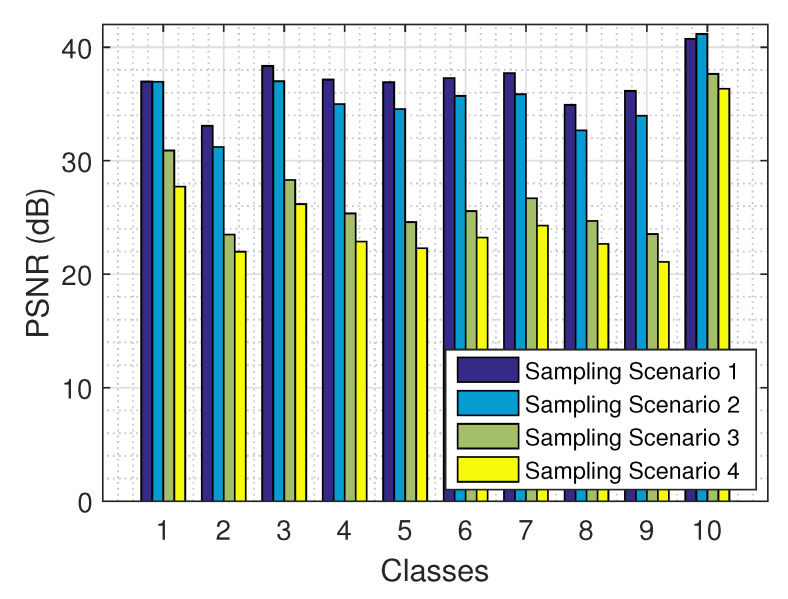
Recovery error for the four missing value scenarios across the ten classes using the logistic model.

The results on the recovery of a tensor from its partial observations are presented in Figure 15 for the 4 sampling scenarios and all 10 classes, using the logistic model. As expected, the larger the amount of the observed values (e.g., in sampling scenario 1), the better the performance of our algorithm. The results are fairly consistent across all 10 classes.

### 4.11. Effect of Missing Values and the Recovery on the Classification Performance

In this set of experiments we are concerned with the scenario where missing measurements occur in environmental sensing data, and we therein explore the beneficial impact of our proposed approach for completing higher-order structural information via tensor modeling on the ensuing classification process. In order to do so, we consider the 4 different missing values scenarios described previously, and we quantify the performance of the trained CNN model in each one of them, in an attempt to illuminate the effect of missing measurements features (e.g., nominal number, spatial size) in the system generalization capability under real-world imperfections.

**Figure 16:**
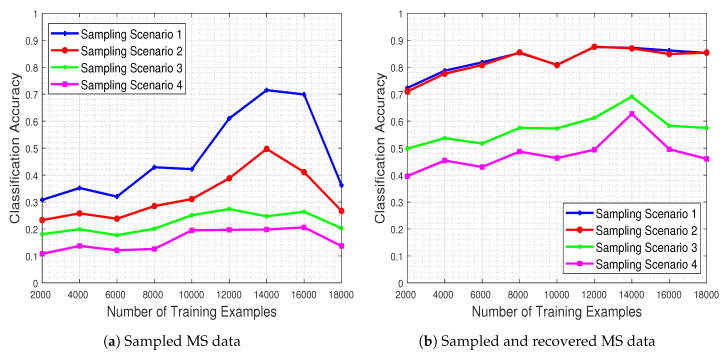
Classification accuracy as a function of the number of training examples for the case of images with missing values and their completed counterparts via our technique. Our recovery algorithm leads to higher levels of classification accuracy for all sampling scenarios

Figure 16 shows the system’s classification performance, in the presence of the missing values (Figure 16a) and when these missing values are recovered by means of our proposed method (Figure 16b). The results demonstrate that the CNN’s classification performance improves dramatically when our missing data recovery algorithm is employed for all four scenarios.

### 4.12. Effect of Missing Values and Quantization on the Recovery

**Figure 17:**
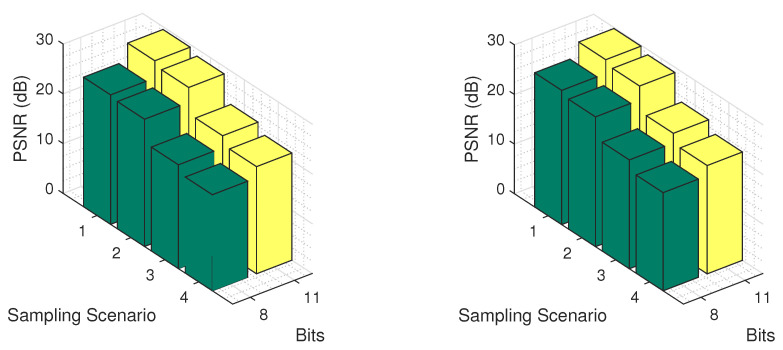
Recovery error for two quantization levels and the four missing value scenarios on classes Pasture (**left**) and Permanent Crop (**right**), using the logistic model.

An interesting issue concerns the recovery of the real values of an image from its partial quantized observations. Therefore, in order to examine the effects of both missing values and quantization in the test images, we applied our method for the four missing value scenarios and for two quantization levels, namely for 8 and 11 bits. The results are presented in Figure 17, for classes *Pasture* and *Permanent Crop*, using the logistic model. As expected, as the missing values increase, the recovery error increases for both quantization levels. Of course, the more the bits of quantization, the better the performance of our recovery.

**Figure 18:**
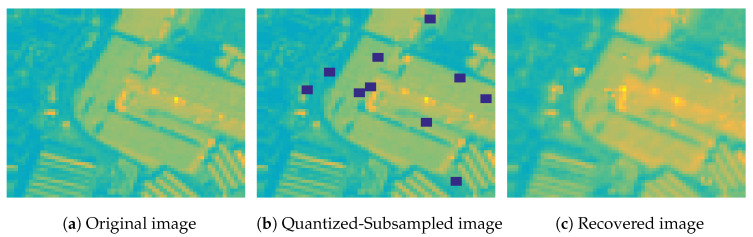
The second spectral band of a MS image from the Industrial class, and the corresponding quantized and subsampled image using 8 bits and sampling scenario 1, as well the recovered image, using the logistic model.

In Figure 18 we show the second spectral band of a MS image from the *Industrial* class, and the corresponding quantized and subsampled image using 8 bits of quantization and sampling scenario 1, as well the recovered image using the logistic model. As we observe, the original MS image can be recovered with high fidelity from partial quantized measurements using our proposed algorithm, despite that the missing elements are not randomly distributed in the image.

### 4.13. Effect of the Quantization & Missing Values on the Classification Performance

To test the system’s performance in the presence of corrupted MS data (i.e., quantized and subsampled), we evaluated the trained CNN model to a test set that has been quantized to a specific number of bits and subsequently subsampled as well.

**Figure 19:**
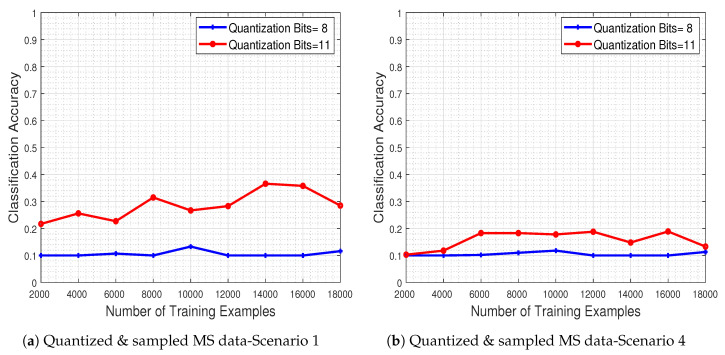

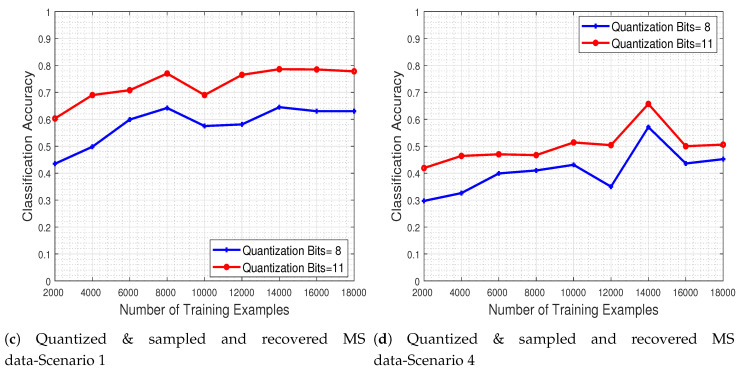
Classification accuracy regarding the number of training examples for indicative number of quantization bits employed in the test set image samples and each different sampling scenario (**a**,**b**), recovered right after the quantization process (**c**,**d**). The system’s performance is heavily affected by the various confronted types of signal degradation, but augmenting the number of bits used for quantization—as well employing the proposed recovery scheme—clearly ameliorates the obtained performance, even when the spatial size and the number of missing measurements is large.

In Figure 19a,b, we depict the obtained performance as a function of the number of training examples, for two quantization levels (8 and 11 bits) and for the two most extreme missing value scenarios (i.e., scenarios 1 & 4). The results clearly show that the system’s classification accuracy is severely degraded due to the quantization and missing value effects. As expected, when more bits are used for the quantization process the performance improves, while increasing the number of missing pixel patches as well as their spatial size adversely affects the classifier.

### 4.14. Effect of the Quantization & Missing Values and the Recovery on the Classification Performance

To demonstrate the ability of the CNN system to classify noisy-infected MS images, we further investigate the effect that our recovery process has on the classification of quantized and subsampled MS images. For that purpose, the trained CNN model is evaluated to a test set that has been recovered via our approach from a quantized and subsampled original image.

Figure 19c,d depict the system’s classification accuracy as a function of the number of training examples, for the recovered by our method versions of test images that were quantized in two levels (with 8 and 11 bits) and for missing value scenarios 1 and 4. The results indicate that adopting a quantization scheme with more bits in conjunction with our proposed recovery process leads to enhanced classification accuracy. Our recovery process clearly improves classification accuracy even in the extreme case where the number of missing pixel patches as well as their spatial size is large.

### 4.15. Joint Effects of Quantization & Missing Values and Recovery on the Classification Performance

**Figure 20:**
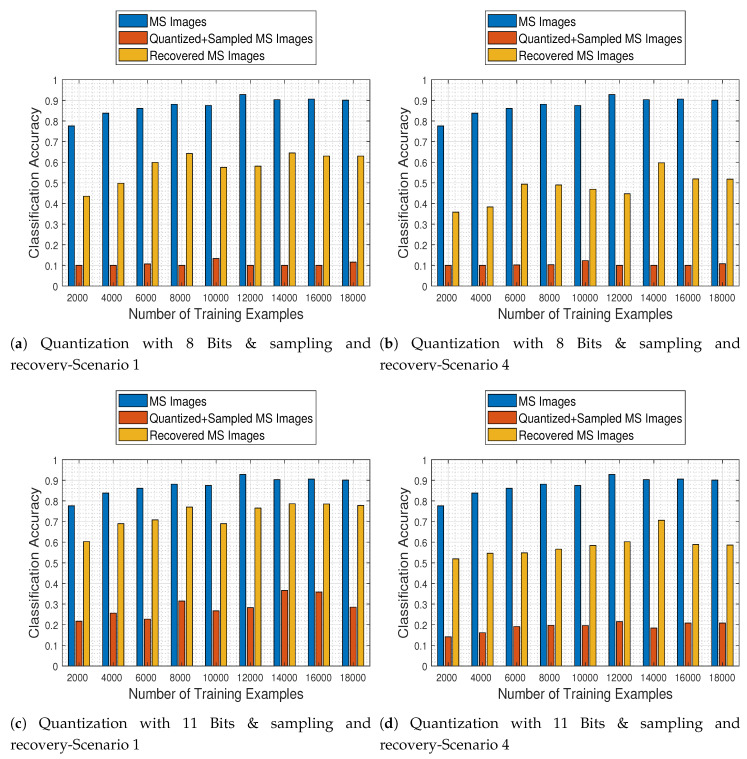
General comparison of quantization with 8–11 Bits &, missing values, and recovery processes on the classification performance.

In the final set of experiments, we conduct a general assessment of our proposed recovery process in order to quantify its effects on the MS image classification task operating on the original images and on the recovered images that were previously quantized and corrupted. Figure 20 depicts the trained CNN performance as a function of the number of image samples it was trained with, when faced with all different kinds of test images (namely original MS, quantized & sampled MS, and recovered MS images), for two quantization levels (8 and 11 bits) and for the two most extreme missing value cases (i.e., scenarios 1 & 4). The results support our claim that the proposed recovery method clearly improves the classification accuracy of the network in all cases, as compared to the CNN being applied directly to the quantized and subsampled images.

### 4.16. Comparison of the Proposed Scheme with Existing Methods

In this section, we compare our approach with other methods which examine the impact of compression on the classification task [45,46,47]. Existing methods apply JPEG2000 on each spectral band, or JPEG2000 followed by DWT or PCA for spectral decorrelation to compress the data. Subsequently, the decompressed-recovered images are fed to standard k-NN or SVM classifiers to perform the supervised ML task. In our system, we compress the MS images by quantization, and we classify the recovered MS images obtained by the proposed recovery algorithm using the pre-trained ResNet-50 model described above.

**Figure 21:**
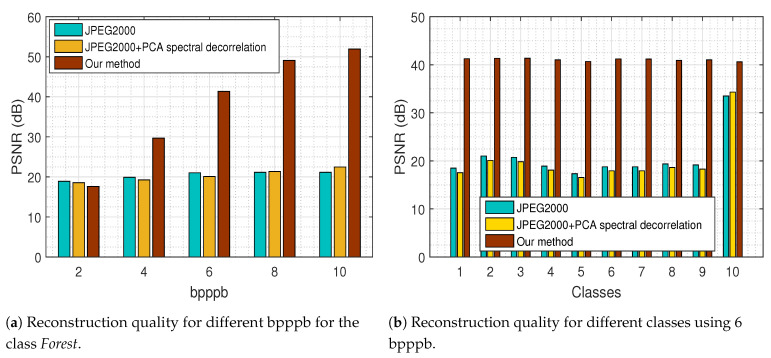
Comparison of our approach with existing compression methods.

At the compression stage of our method, we compared the recovered MS images via the proposed tensor recovery algorithm with the decompressed images via JPEG2000 on each spectral band and via JPEG2000 followed by PCA for spectral decorrelation. The recovery error as a function of *bits per pixel per band* (bpppb) for the class *Forest* presented in Figure 21a, indicates the efficacy of our method since the PSNR increases as the bpppb value gets higher in contrast to the other compression methods that exhibit a fairly constant PSNR behavior even for large values of bpppb.

In addition, we can observe from the results that our approach outperforms existing compression methods, especially for large values of bpppb, as it is demonstrated in Figure 21b for the HS images of each class, using 6 bpppb.

The classification stage is implemented in two levels: one operating on the original MS images and one operating on the recovered MS images. In order to perform a comparison between the adopted ResNet-50 model architecture at both levels, a competing ML model must be selected at first.

Although existing methods studying the compression impact on classification [45,46,47] adopt specific classifiers such as k-NN or SVM, the examined problem therein is of a different nature: pixel-level classification of a MS image (e.g., for object detection purposes) in contrast to scene classification in our case (i.e., the whole image is classified). Furthermore, following the traditional ML pipeline, feature extraction and feature selection stages must take place before the classification stage. If this is not the case, and one simply treats the MS imagery at hand as “long” feature vectors (of pixels) to be fed to the classifier (e.g., an SVM), the whole task is automatically put in jeopardy mainly for two reasons: possible correlations among the data are not exploited as long as no descriptive features are extracted from them, while the computational burden of such a process may be intractable (these classifiers do not perform computations on GPUs as DL-based CNNs).

Based on the aforementioned reasoning, we did not compare the adopted network with standard ML classifiers, but rather with a CNN model trained from scratch. Apart from the ease of GPU computations, adopting such an approach passes the feature extraction stage directly to the model through its training process.

Concerning the architecture of the competing CNN, we adapt a solution involving 3D-CNNs (for tackling human action recognition [62] and video quality assessment [63] problems) to their 2D counterparts for our task at hand. More specifically, the network architecture comprises multiple stacks of convolutional, max-pooling, normalization, and activation (i.e., ReLU) layers. The number of stacks of layers employed was 5, where each convolutional layer is always followed by a max-pooling layer, a batch-normalization layer and an activation layer in that order. These layers are then followed by 2 fully connected layers, and a final soft-max layer for the prediction task.

Based on the findings of the aforementioned reference papers (as well as on our own cross-validation results) we employed small receptive fields (e.g., 3×3) for the convolutional filters, rather than larger ones (e.g., 5×5). As far as the number of filters is concerned, we adopted a “doubling-depth” strategy where the filters are doubled from a previous layer to the next one, starting from 32 filters in the first layer and ending up with 512 in the last layer in that way. All max-pooling layers have a size of 2×2, which corresponds to reducing the input data by a factor of 4, while the fully connected layers have 512 and 256 units respectively. For fairness of comparison, the Adagrad optimizer was selected along with the categorical cross-entropy as the loss function.

**Figure 22:**
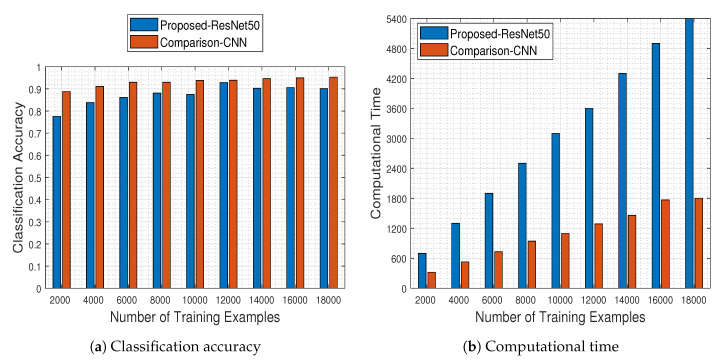
Classification accuracy and respective computational time regarding the number of training examples, for competing classification models. The “lighter” comparison-CNN model outperforms the pre-trained ResNet-50 model by an accuracy margin of up to 2.5% in the best case, being faster at the same time.

Being aligned with the results presented in Figure 6, in Figure 22 we depict the respective results obtained by the *“comparison-CNN”* model described above.

As can be seen in Figure 22a,b, the comparison-CNN model outperforms the pre-trained ResNet-50 model for all cases of training example sizes, a fact that can be attributed mainly to 2 reasons:

- The pre-trained ResNet-50 model was originally designed for RGB image recognition (and not for MS one), where its success rate was over 98.5% (i.e., in the EUROSAT RGB dataset). Adapting it appropriately led to a logical performance drop to 92.8%, which was to be expected since the problem is of a *similar* flavor and not exactly the same. In contrast, the comparison-CNN reaches a success rate of up to 95.3%, indicating that models designed for video processing purposes can scale back well to image processing tasks.
- The comparison-CNN model is quite faster than the pre-trained ResNet-50 one, a fact which can be attributed to the number of trainable parameters of each architecture. To that end, in Table 3 we present the parameters that must be learned by each model.
  Of course, as long as the comparison-CNN model was approximately 12 times “lighter” than the ResNet-50 one, the computational time needed for its training was expected to be less, as shown in Figure 22b.

**Figure 23:**
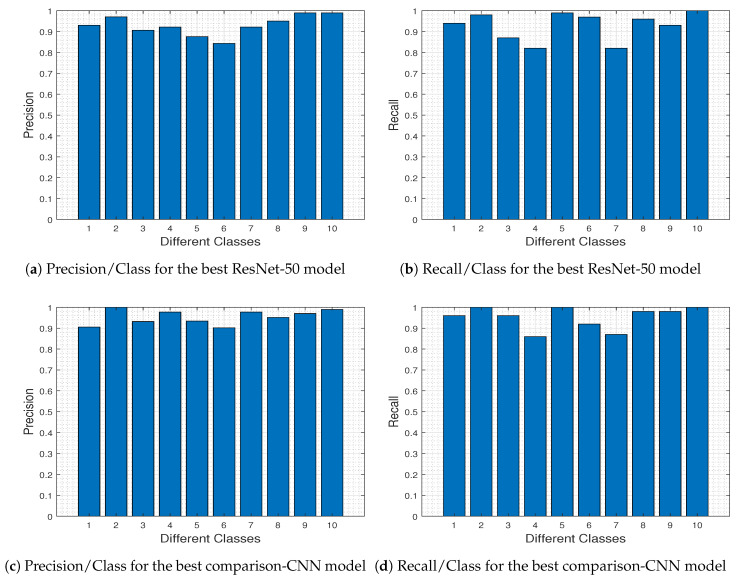
Precision and recall plots per each different class for the pre-trained ResNet-50 (**top**) and comparison-CNN (**bottom**).

To further investigate the specific differences between the two competing DL models, in Figure 23 we depict the precision-recall metrics per class obtained by the ResNet-50 model (Figure 23a,b) and the comparison-CNN model (Figure 23c,d).

In ML and *Receiver Operating Characteristics* (ROC) analysis [64], *Recall*/*Sensitivity*/*True Positive Rate* refers to the *proportion of Real Positive cases that are correctly Predicted Positive*. In contrast, *Precision*/*Confidence*/*Positive Predicted Value* denotes the *proportion of Predicted Positive cases that are correctly Real Positives*. Comparing Figure 23a–c and Figure 23b–d, we 0 outperforms ResNet-50 for classes 3, 4, and 6 (i.e., Herbaceous Vegetation, Highway and Permanent Crop, respectively). These classes are the most difficult to predict using the pre-trained ResNet-50 model (as dictated from Figure 8, and discussed therein), and this difficulty explains the fact that the comparison-CNN exhibits an increased accuracy.

**Figure 24:**
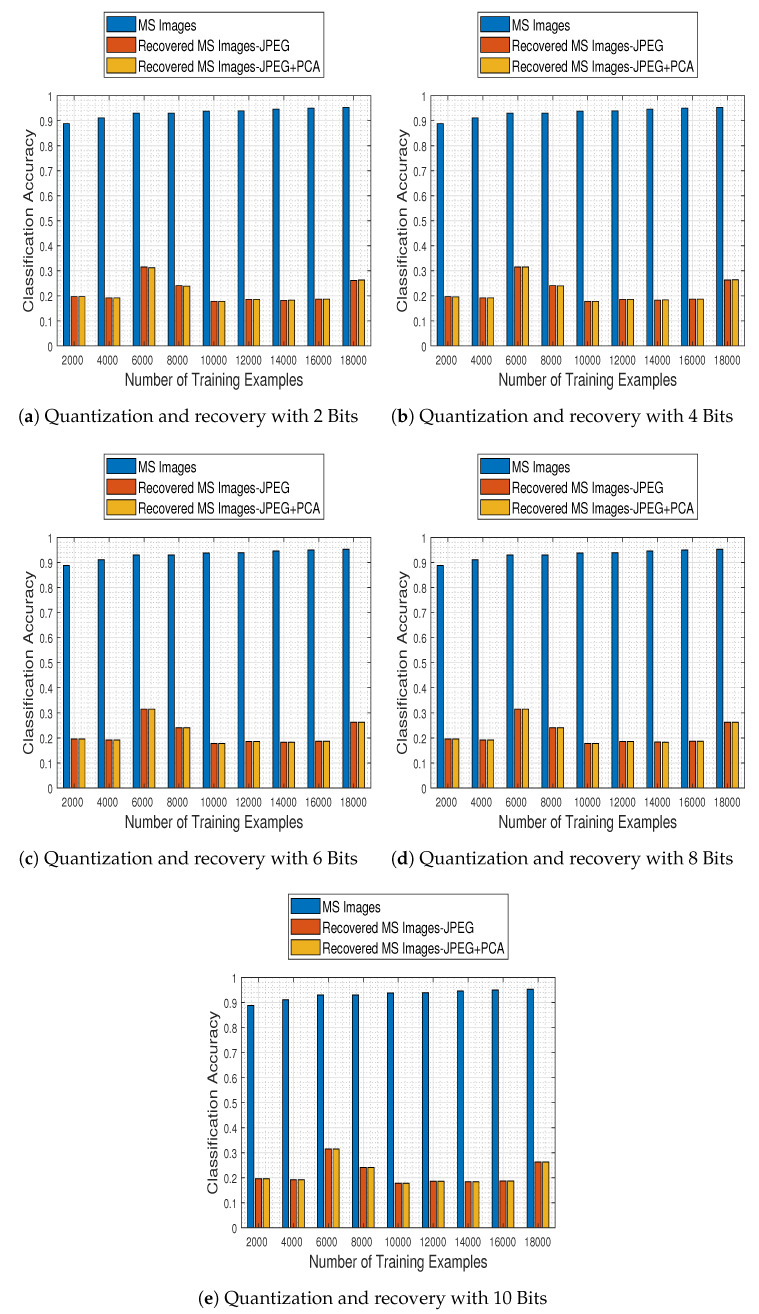
Comparison of the quantization and the recovery processes on the classification performance, when competing quantization schemes (JPEG, JPEG+PCA) are adopted.

Although the comparison results described above concern the first level of the classification stage, the most important ones concern the second level, in which the trained model is used in conjunction with the compression stage method adopted. To that end, we selected the best model of the comparison-CNN described above (i.e., the one obtained when trained with 18,000 training examples and reaching 95.3% accuracy) and investigated the effect of compression and recovery via the two schemes mentioned earlier: JPEG2000 and JPEG2000 followed by PCA across the spectral dimension. In Figure 24 we depict the obtained results for both methods (JPEG stands for JPEG2000), in contrast to those obtained for the compression-free situation portrayed earlier.

The results demonstrate that the alternative quantization schemes have a devastating effect in the system’s performance, which—irrespective to the number of bits to which the images are quantized to—remains nearly fixed to a maximum accuracy of up to 31.5%.

Based on that remark, we subsequently performed the final and most crucial system-to-system comparison, between our proposed one (i.e., ResNet-50 model and tensor recovery) and the competing ones (comparison-CNN and JPEG200 & JPEG200+PCA).

**Figure 25:**
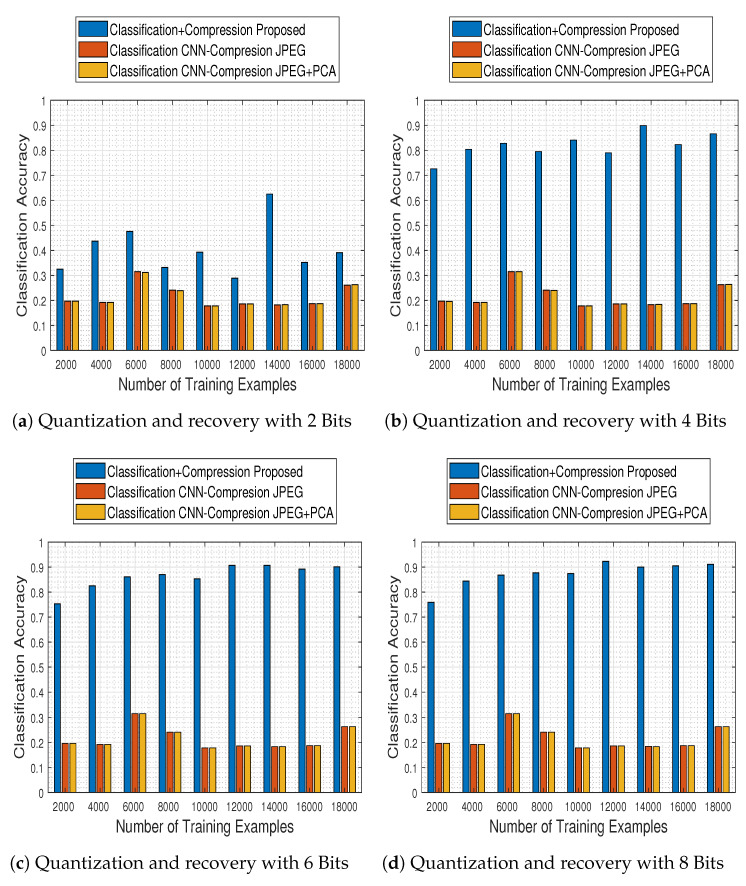

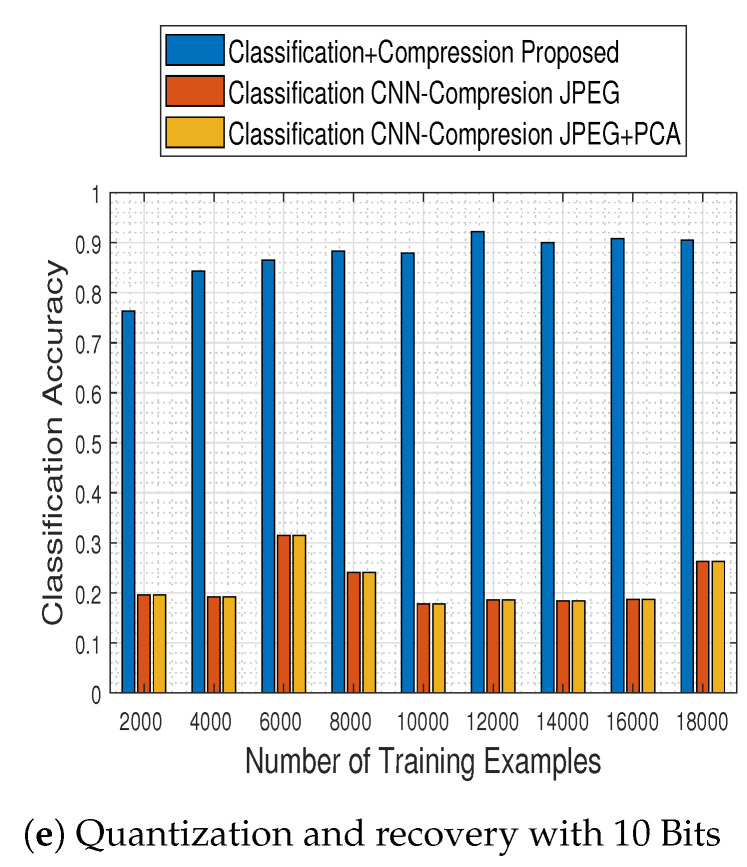
General system comparison of the quantization-recovery-classification processes. The proposed system clearly outperforms both competing ones in every examined quantization bits scenario.

Figure 25 contains classification accuracy results obtained by our system as well as the competing ones, with respect to the number of training examples with which the classification schemes were trained with and the number of bits to which the compression schemes quantized the MS images.

The results clearly imply that the proposed system not only outperforms the competing ones in every examined case, but its performance steadily ameliorates as well. In contrast, its competitors at the same time obtain poor performance results in every case, indicating in that way the efficacy of the proposed approach.

## 5. Conclusions

In this work, we proposed a DL architecture for the problem of classifying MS image data. The approach is based on residual networks, successfully used for the respective RGB task for learning efficient spatial feature representations. Furthermore, we designed, tested, and evaluated with actual data two novel techniques to address the real-life scenarios of dealing with quantized data and missing values in the images. A constrained maximum likelihood estimation on the tensor unfoldings is introduced for the recovery of the real values, taking into account the quantization bin boundaries and the existence of possible noise. Based on our experimental findings on a recently released—sufficiently large—MS image dataset, we demonstrated that improved classification accuracy is feasible even in the presence of only half of available data observations using state-of-the-art CNNs. Clearly, adopting a tensor recovery algorithm proved to be a good strategy for efficient classification of previously quantized, as well as subsampled MS data demonstrating the efficacy of the proposed approach.

## Figures and Tables

**Table 1:** Dataset split among training-validation-test sets. Each class is split into 90%-5%-5% training-validation-test sets respectively, ending up with a training set of 18,000 samples and validation & test sets of 1000 samples each.

Class Name	Available Samples	Training Set Samples	Validation Set Samples	Test Set Samples
Annual Crop	2000	1800	100	100
Forest	2000	1800	100	100
Herbaceous Vegetation	2000	1800	100	100
Highway	2000	1800	100	100
Industrial	2000	1800	100	100
Pasture	2000	1800	100	100
Permanent Crop	2000	1800	100	100
Residential	2000	1800	100	100
River	2000	1800	100	100
Sea Lake	2000	1800	100	100
**Total**	**20,000**	**18,000**	**1000**	**1000**

**Table 2:** Recovery error for different compression ratios on the MS images of each class, using the logistic model.

PSNR (dB)	Compression Ratio			
	×6.9	×3.4	×2.25	×1.67
Annual Crop	18.14	29.60	41.25	47.34
Forest	17.59	29.68	41.34	49.09
Herbaceous Vegetation	18.04	29.50	41.37	48.81
Highway	17.76	29.48	41.02	47.82
Industrial	17.56	29.40	40.68	47.11
Pasture	18.09	29.75	41.22	47.73
Permanent Crop	18.08	29.55	41.19	47.82
Residential	17.87	29.46	40.90	48.04
River	18.04	29.61	41.02	48.15
Sea Lake	21.74	30.84	40.62	52.80

**Table 3:** Number of trainable parameters of the competing DL models. A more complex network architecture clearly leads to a more computational thirsty model.

Network Name	Number of Trainable Parameters
ResNet-50	23,586,442
Comparison-CNN	1,969,994

## Abbreviations

The following abbreviations are used in this manuscript:

RS	Remote Sensing
MS	Multispectral
HS	Hyperspectral
DWT	Discrete Wavelet Transform
ML	Machine Learning
SVM	Support Vector Machine
RF	Random Forest
DL	Deep Learning
CNN	Convolutional Neural Network
LEOP	Launch and Early Orbit Phase
k-NN	k-Nearest Neighbors
PCA	Principal Component Analysis
NN	Neural Network
ILSVRC	ImageNet Large-Scale Visual Recognition Challenge
SGD	Stochastic Gradient Descent
CDF	Cumulative Distribution Function
SVD	Singular Value Decomposition
PSNR	Peak-Signal-to-Noise-Ratio
MSE	Mean Square Error
bpppb	bits per pixel per band
ROC	Receiver Operating Characteristics
